# Bidirectional association between inflammatory bowel disease and type 1 diabetes: a nationwide matched cohort and case-control study

**DOI:** 10.1016/j.lanepe.2024.101056

**Published:** 2024-08-31

**Authors:** Jiangwei Sun, Jialu Yao, Ola Olén, Jonas Halfvarsson, David Bergman, Fahim Ebrahimi, Sofia Carlsson, Johnny Ludvigsson, Jonas F. Ludvigsson

**Affiliations:** a Department of Medical Epidemiology and Biostatistics, Karolinska Institutet, Stockholm, Sweden; b Clinical Epidemiology Division, Department of Medicine Solna, Karolinska Institutet, Stockholm, Sweden; c Sachs’ Children and Youth Hospital, Stockholm South General Hospital, Stockholm, Sweden; d Department of Clinical Science and Education Södersjukhuset, Karolinska Institutet, Stockholm, Sweden; e Department of Gastroenterology, Faculty of Medicine and Health, Örebro University, Örebro, Sweden; f Department of Gastroenterology and Hepatology, University Digestive Health Care Center Basel – Clarunis, Basel, Switzerland; g Institute of Environmental Medicine, Karolinska Institutet, Stockholm, Sweden; h Crown Princess Victoria’s Children’s Hospital, Region Östergötland, Linköping, Sweden; i Division of Paediatrics, Department of Biomedical and Clinical Sciences, Linköping University, Linköping, Sweden; j Department of Pediatrics, Örebro University Hospital, Örebro, Sweden; k Division of Digestive and Liver Disease, Department of Medicine, Columbia University Medical Center, New York, USA

**Keywords:** Inflammatory bowel disease, Type 1 diabetes, Cohort, Case–control, Nationwide

## Abstract

**Background:**

Co-occurrence of inflammatory bowel disease (IBD) and type 1 diabetes (T1D) has been linked to poor clinical outcomes, but evidence on their bidirectional associations remain scarce. This study aims to investigate their bidirectional associations.

**Methods:**

A nationwide matched cohort and case–control study with IBD patients identified between 1987 and 2017. The cohort study included 20,314 IBD patients (≤28 years; Crohn’s disease [CD, n = 7277], ulcerative colitis [UC, n = 10,112], and IBD-unclassified [IBD-U, n = 2925]) and 99,200 individually matched reference individuals, with a follow-up until December 2021. The case–control study enrolled 87,001 IBD patients (no age restriction) and 431,054 matched controls. We estimated adjusted hazard ratio (aHR) of incident T1D in the cohort study with flexible parametric survival model and adjusted odds ratio (aOR) of having a prior T1D in the case–control study with conditional logistic regression model, with 95% confidence intervals (CI).

**Findings:**

During a median follow-up of 14 years, 116 IBD patients and 353 reference individuals developed T1D. Patients with IBD had a higher hazard of developing T1D (aHR = 1.58 [95% CI = 1.27–1.95]). The hazard was increased in UC (aHR = 2.02 [1.51–2.70]) but not in CD or IBD-U. In the case–control study, a total of 1018 (1.2%) IBD patients and 3496 (0.8%) controls had been previously diagnosed with T1D. IBD patients had higher odds of having prior T1D (aOR = 1.36 [1.26–1.46]). Such positive association was observed in all IBD subtypes. The sibling comparison analyses showed similar associations between IBD and T1D (aHR = 1.44 [0.97–2.15] and aOR = 1.32 [1.18–1.49]).

**Interpretation:**

Patients with IBD had a moderately increased hazard of developing T1D and higher odds of having prior T1D. Their bidirectional associations may be partially independent of shared familial factors.

**Funding:**

10.13039/100018353European Crohn’s and Colitis Organisation, 10.13039/501100009800Stiftelsen Professor Nanna Svartz Fond, 10.13039/501100003748SSMF (project#: PG-23-0315-H-02), 10.13039/501100009782Ruth and Richard Julin Foundation; and 10.13039/501100006636FORTE (project#: 2016-00424).

**Research in context**

**Evidence before this study**

We searched PubMed and Web of Science for studies on the association between inflammatory bowel disease (IBD) and type 1 diabetes (T1D), from database inception to April 30, 2024, with a combination of the following keywords ((inflammatory bowel disease OR IBD OR Crohn’s disease OR ulcerative colitis) AND (type 1 diabetes OR T1D) AND (observational study OR cohort OR case–control study)) and no language restriction. Overall, despite possibly shared pathological pathways and co-occurrence of IBD and T1D leading to poor clinical outcomes, there is no conclusive evidence on the bidirectional association between IBD and T1D. Moreover, given the familial aggregation of IBD and T1D, whether the previously observed associations were due to shared familial factors remains unclear.

**Added value of this study**

In this nationwide matched cohort and case–control study, patients with IBD had a higher hazard of developing T1D and higher odds of having prior T1D than the general population. The bidirectional association between them may be partially independent of shared genetics and early environmental factors and was more pronounced in adult-onset IBD patients, males, patients with ulcerative colitis, and those with primary sclerosing cholangitis.

**Implications of all the available evidence**

Given IBD and T1D have not been addressed as comorbidities in current management guidelines, awareness of the increased co-occurrence risk may facilitate early detection of both conditions. Moreover, physicians should actively consider measuring blood glucose or HbA1c in IBD patients with unexplained weight loss or fatigue to check for diabetes.

## Introduction

Inflammatory bowel disease (IBD) is a chronic inflammatory gastrointestinal (GI) disorder that affects 0.5–1% of individuals in the world.^1^, ^2^, ^3^ Subtypes of IBD include Crohn’s disease (CD), ulcerative colitis (UC), and IBD-unclassified (IBD-U, subtype uncertainty). Type 1 diabetes (T1D) is an autoimmune disease characterized by a destruction of pancreatic β-cells that leads to lifelong absolute insulin deficiency. While T1D affects ∼0.1% of the global population, its prevalence in Sweden is about 0.5%.^4^^,^^5^ Intricate interplays among immune hyperactivation, metabolic perturbation, and gut microbiota disturbance have been suggested in the pathogenesis of both IBD and T1D.^6^, ^7^, ^8^

Despite possibly shared pathological pathways, there is inconclusive evidence on the association between IBD and T1D. Genome-wide association studies suggested that genetic loci shared between IBD and T1D had mixed effects on the development of these diseases.^9^ Results from epidemiological studies have also been inconsistent (Supplementary Table S1). A meta-analysis that summarized cross-sectional and case–control studies from 2007 through 2019 suggested no association between IBD and T1D but with substantial heterogeneity.^10^ Later individual studies (including three cohort studies and one cross-sectional study) reported a positive association in European populations,^11^, ^12^, ^13^, ^14^ while the increased T1D risk in Korean IBD patients failed to reach statistical significance.^15^ Besides, previous studies have limitations including not adjusting for potential confounders (e.g., other autoimmune comorbidities)^11^, ^12^, ^13^^,^^15^ and not investigating the bidirectional association between IBD and T1D. Moreover, few studies have failed to consider potential confounding from familial factors (i.e., shared genetics and early environmental factors), which is an important limitation due to the familial aggregation of IBD or T1D and a mixed effect of some genetic variants.^9^^,^^16^^,^^17^

We therefore conducted a nationwide matched cohort and case–control study to investigate the bidirectional association between IBD and T1D by using Swedish national registers. Given shared pathological pathways,^1^^,^^2^^,^^4^ we hypothesized that individuals with IBD had increased risks of both developing T1D and having a prior diagnosis of T1D. In addition, sibling comparison analyses were performed to account for shared familial factors.^18^

## Methods

### Data source

**Fig. 1:**
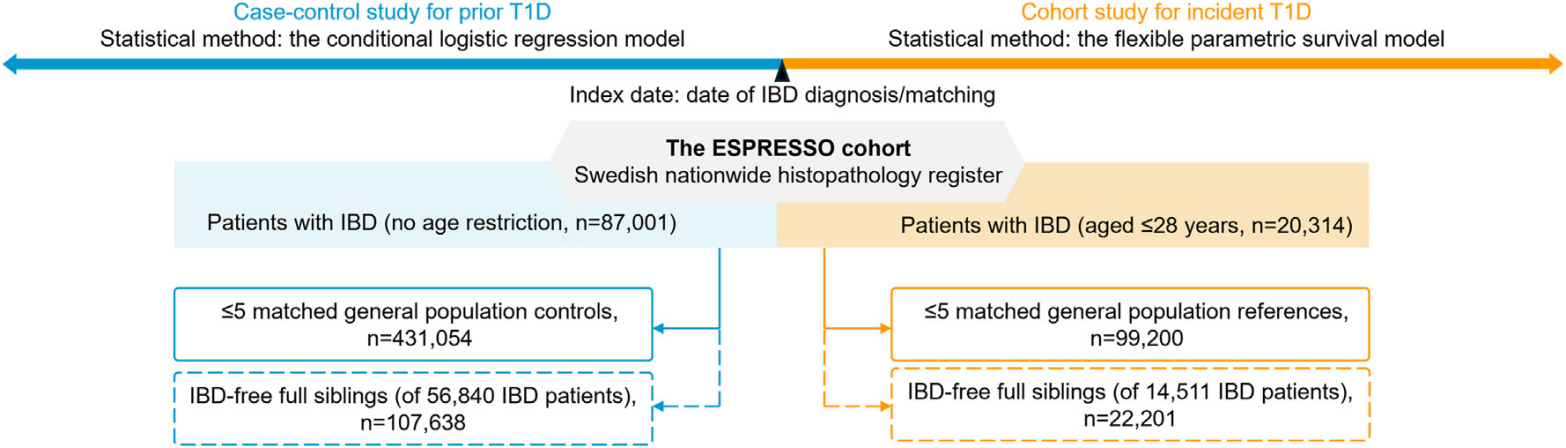
Diagram of the study design for the bidirectional association between inflammatory bowel disease (IBD) and type 1 diabetes (T1D). ESPRESSO: Epidemiology Strengthened by histoPathology Reports in Sweden.

This study was based on a nationwide histopathology cohort ESPRESSO [Epidemiology Strengthened by histoPathology Reports in Sweden] and the Swedish National Patient Register (NPR). ESPRESSO contains gastrointestinal biopsies from all 28 pathology departments in Sweden from 1965 to 2017.^19^ Computerized information included the date of biopsy, topography, and morphology (by SNOMED [the Swedish version of the Systematized Nomenclature of Medicine]). The NPR started in 1964 and covers nationwide inpatient care since 1987 and outpatient care since 2001.^20^ Individuals were linked across Swedish national healthcare registers via the unique personal identity number,^21^ which enables a virtually complete follow-up in the cohort study. The diagram of the study design is shown in Fig. 1.

### Identification of IBD patients and comparison groups

We identified patients with IBD with ≥1 International Classification of Disease (ICD) code for IBD in the NPR and ≥1 IBD-indicative biopsy in ESPRESSO (see Supplementary Table S2 for IBD definitions). This approach had a positive predictive value (PPV) of 95% for IBD diagnosis.^22^^,^^23^ To avoid immortal time bias, the date of IBD diagnosis (i.e., the index date) was defined as the date of fulfilling both criteria. We also identified different phenotypes of IBD from the NPR using the Montreal classification criteria.^24^ This included CD location and perianal disease modifier, UC extent, and presence of primary sclerosing cholangitis (PSC) as well as other extraintestinal manifestations (see Supplementary Table S3 for ICD codes defining IBD phenotypes).

There were two comparison groups (Fig. 1). In the general population comparison group, up to five reference individuals from the Total Population Register (TPR)^25^ were matched with each IBD patient by birth year, sex, county of residence, and calendar year. In the IBD-free full siblings comparison group, full siblings of IBD patients were involved to assess the influence of residual confounding from shared genetics and early environmental factors shared within families.^18^ They were identified from the Swedish Multi-Generation Register,^26^ a component of the TPR. The reference individuals and full siblings had to be alive, living in Sweden, and free of IBD and T1D when being selected (i.e., index date).

### Ascertainment of T1D

T1D diagnosis was identified from the NPR based on ICD codes (see Supplementary Table S4a for T1D definitions). Because ICD-8 and ICD-9 cannot distinguish type 1 and type 2 diabetes until ICD-10, we required the individuals to be aged ≤30 years when identifying the T1D diagnosis via ICD-8 or ICD-9. Such criteria have been validated with a satisfactory PPV of 95%.^27^

### Matched cohort for IBD and incident T1D

In the cohort study for IBD and incident T1D, we only enrolled IBD patients aged ≤28 years with a diagnosis of IBD from January 1, 1987, through December 31, 2017 (Fig. 1). The baseline age was chosen to allow a window of at least 2 years for new-onset T1D. Individuals who had been diagnosed with T1D or gestational diabetes before the index date were excluded (see Supplementary Table S4a for exclusion criteria). We followed the enrolled population from the index date until T1D diagnosis, emigration, gestational diabetes, diabetes mellitus other than T1D, death, or 31 December 2021. Individuals from the two comparison groups were censored if being diagnosed with IBD during the follow-up period.

### Matched case–control study for IBD and prior T1D

In the matched case–control study, we enrolled IBD cases (no restriction on IBD diagnosis age) diagnosed from January 1, 1987, through December 31, 2017, and general population matched by birth year, sex, county of residence, and calendar year as controls (Fig. 1). Full siblings of IBD patients were also enrolled.

### Covariates

We considered the following covariates in addition to matching variables (i.e., birth year, sex, county of residence, and calendar year). Country of birth (Nordic [including Sweden, Denmark, Finland, Norway, and Iceland] or others) was identified from the Total Population Register.^25^ Educational attainment (four groups: 0–9 years, 10–12 years, ≥13 years, and “missing”) was identified from the Swedish Longitudinal Integrated Database for Health Insurance and Labor Market Studies (LISA)^28^ and used as a proxy for socioeconomic status. For individuals aged <18 years, the highest educational level of their parents, also identified from LISA, was used. Data on other autoimmune diseases before the index date were also collected from the NPR (see Supplementary Table S4a for ICD codes).

### Statistical analyses

Relative risk estimates were reported as hazard ratio (HR) in the cohort study and odds ratio (OR) in the case–control study. In the matched cohort study, we applied the flexible parametric survival model to calculate the HR with 95% confidence interval (CI).^29^^,^^30^ This method allowed the effect of IBD to be time-varying. We explored the association for overall IBD and then for IBD subtypes (i.e., CD, UC, and IBD-U). We treated competing events (e.g., death) as censoring events. In the matched case–control study, conditional logistic regression models were fitted to estimate the OR with 95% CI.^31^ Two models were used in both cohort and case–control studies. We conditioned the analyses on matching variables (i.e., birth year, sex, county of residence, and calendar year) in model 1, and additionally adjusted for country of birth, educational attainment, and history of any other autoimmune disease in model 2. In the sibling comparison analyses, we calculated risk estimates based on all covariates in model 2 plus an additional family identifier.

### Subgroup and sensitivity analysis

In both cohort and case–control studies, we calculated stratum-specific relative risks by sex (female/male), age at index date, calendar period of IBD diagnosis/matching date (1987–2001, 2002–2009, or 2010–2017), educational attainment (0–9, 10–12, ≥13 years, or “missing”), history of other autoimmune diseases (yes/no), and different IBD phenotypes at baseline, including CD location and perianal disease modifier, UC extent, presence of PSC, and other extraintestinal manifestations.

Several sensitivity analyses were conducted to test the robustness of our findings. **First,** to assess the potential influence of detection bias (i.e., work up for one disease increases the chance of diagnosing another one), surveillance bias (i.e., regular check-ups after one disease’s diagnosis increases the chance of early detection of another one), and reverse causation (due to the pre-clinical stage and delayed diagnosis of IBD or T1D) on the studied association, we discarded the first one and then three years of follow-up from the cohort study and omitted T1D diagnosed within one and three years respectively *before* IBD diagnosis in the case–control study. **Second,** in the cohort study, to estimate the influence of T1D definition, we identified T1D patients as those with the primary diagnosis or those with at least two diagnoses. **Third,** we restricted the analysis to those with data on educational attainment (1990 onward). **Fourth,** to avoid potential misclassification of T1D with other conditions, we additionally censored the follow-up on diagnoses of diabetes insipidus, nephrogenic diabetes, pancreatitis, pancreatic insufficiency, and pancreatic cancer (see Supplementary Table S4a for ICD codes). **Fifth,** to rule out the potential impact of IBD treatments (including IBD-related surgery, steroids, and biologics, see Supplementary Table S4b and 4c for related codes) on the studied associations, we further censored patients at exposure to each of these treatments. In the analysis for IBD surgery, we censored the follow-up at the date of first IBD-related surgery. In the analysis involving steroids and biologics, we limited the analysis to individuals with an index date of January 2006 or later and censored the follow-up at the date of first prescription of steroid or biological therapy after IBD diagnosis, which were identified from the Prescribed Drug Register (available since July 2005).^32^ **Sixth,** to investigate the potential influence of residual confounding from shared genetics and early environmental factors, we compared patients with IBD with their IBD-free full siblings.

Data analyses were performed using SAS (version 9.4; SAS Institute Inc, Cary, NC), Stata (version 16.1; StataCorp LP, College Station, TX), and R (version 3.6.0). A two-sided p ≤ 0.05 was considered statistically significant.

### Ethics

This study was approved by the Stockholm Ethics Review Board (2014/1287-31/4, 2018/972-32, and 2022-05774-02). Individual informed consent was waived as the study was register-based.

### Role of the funding source

The funders of the study had no role in study design, data collection, data analysis, data interpretation, or writing of the report.

## Results

### Baseline characteristics of the cohort study

A total of 20,314 patients with IBD (CD: 7277; UC: 10,112; IBD-U: 2925) and 99,200 matched reference individuals were enrolled in the analyses. Patients with IBD had a median age of 20.8 years at index date (33.0% diagnosed <18 years) and females comprised 46.4% of IBD patients. The prevalence of autoimmune diseases other than T1D was higher in IBD patients (9.0%) than in reference individuals (3.8%) (Table 1). PSC was found in 1.4% of IBD patients, colonic location in 14.1% of CD patients, and extensive colitis in 20.2% of UC patients (Supplementary Table S5).

**Table 1:** Characteristics of patients with IBD and their matched reference individuals in the cohort study, n (%).

	References	Patients with IBD	Subtypes of IBD		
			CD	UC	IBD-U
N	99,200	20,314	7277	10,112	2925
Age at index date, years^a^					
Mean ± SD	19.9 ± 5.4	20.0 ± 5.4	19.6 ± 5.4	20.6 ± 5.3	19.4 ± 5.8
Median (IQR)	20.7 (16.4–24.3)	20.8 (16.5–24.5)	20.1 (15.8–24.0)	21.5 (17.3–24.8)	20.0 (15.7–24.1)
<18	33,268 (33.5)	6700 (33.0)	2697 (37.1)	2873 (28.4)	1130 (38.6)
18–≤28	65,932 (66.5)	13,614 (67.0)	4580 (62.9)	7239 (71.6)	1795 (61.4)
Female	45,940 (46.3)	9431 (46.4)	3575 (49.1)	4480 (44.3)	1376 (47.0)
Born in Nordic country	88,265 (89.0)	18,858 (92.8)	6660 (91.5)	9462 (93.6)	2736 (93.5)
Calendar period at index date^a^					
1987–2001	31,031 (31.3)	6355 (31.3)	2368 (32.5)	3442 (34.0)	545 (18.6)
2002–2009	33,533 (33.8)	6865 (33.8)	2417 (33.2)	3490 (34.5)	958 (32.8)
2010–2017	34,636 (34.9)	7094 (34.9)	2492 (34.2)	3180 (31.5)	1422 (48.6)
Educational attainment, years					
0–9	24,035 (24.2)	4683 (23.1)	1837 (25.2)	2133 (21.1)	713 (24.4)
10–12	44,860 (45.2)	9364 (46.1)	3284 (45.1)	4763 (47.1)	1317 (45.0)
≥13	26,706 (26.9)	5648 (27.8)	1888 (25.9)	2906 (28.7)	854 (29.2)
Missing	3599 (3.6)	619 (3.1)	268 (3.7)	310 (3.1)	41 (1.4)
History of autoimmune diseases	3720 (3.8)	1822 (9.0)	931 (12.8)	557 (5.5)	334 (11.4)
Follow-up time, years					
Median (IQR)	14.3 (9.0–20.8)	14.6 (9.2–20.8)	14.9 (9.3–21.2)	15.3 (9.7–21.1)	11.8 (8.0–17.4)
0–0.9	725 (0.7)	101 (0.5)	41 (0.6)	48 (0.5)	12 (0.4)
1–4.9	4080 (4.1)	630 (3.1)	213 (2.9)	273 (2.7)	144 (4.9)
5–9.9	24,981 (25.2)	5112 (25.2)	1812 (24.9)	2339 (23.1)	961 (32.9)
10–19.9	41,482 (41.8)	8706 (42.9)	3036 (41.7)	4350 (43.0)	1320 (45.1)
≥20	27,932 (28.2)	5765 (28.4)	2175 (29.9)	3102 (30.7)	488 (16.7)

CD: Crohn’s disease; IBD(-U): inflammatory bowel disease (unclassified); IQR: interquartile range; SD: standard deviation; UC: ulcerative colitis.

a Index date: date of IBD diagnosis for patients with IBD, and date of selection for their matched population reference individuals.

### IBD and the hazard of incident T1D

**Fig. 2:**
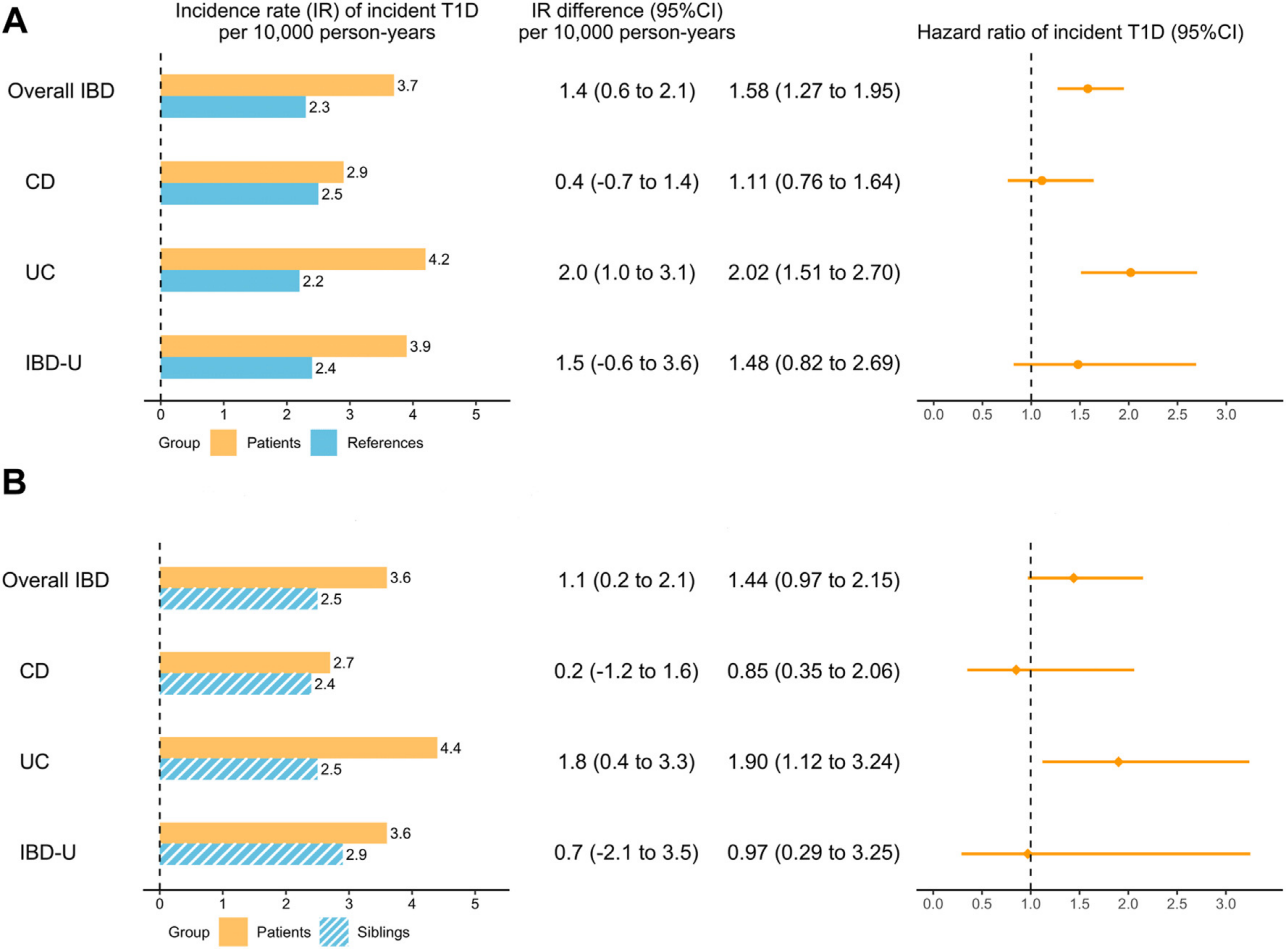
Incidence rate (IR) and hazard ratios for incident type 1 diabetes (T1D) in patients with inflammatory bowel disease (IBD), compared with their matched reference individuals (A) and full siblings (B), with 95% confidence interval (CI). The hazard ratio was estimated from the flexible parametric survival model in Model 2. CD: Crohn’s disease; IBD-U: IBD-unclassified; and UC: ulcerative colitis.

During a median follow-up of around 14 years (>70% individuals followed for >10 years), incident T1D was observed in 116 (0.6%) patients with IBD (CD: 33; UC: 68; IBD-U: 15) and in 353 (0.4%) reference individuals, with an incidence rate (IR) difference of 1.4/10,000 person-years (95% CI: 0.6–2.1; IR in two groups: 3.7 vs. 2.3 per 10,000 person-years). The median (interquartile range: IQR) age of T1D diagnosis was 27.5 (20.8–35.3) and 27.2 (21.2–33.6) in IBD patients and the reference individuals, respectively. After multivariable adjustment, patients with IBD had a higher hazard of developing T1D (aHR = 1.58 [95% CI: 1.27–1.95]) (Fig. 2 & Supplementary Table S6) than the reference individuals. The increased hazard was driven by UC (aHR = 2.02 [1.51–2.70]), but not CD (aHR = 1.11 [0.76–1.64]) or IBD-U (aHR = 1.48 [0.82–2.69]).

### Subgroup and sensitivity analyses in the cohort study

The increased hazard of T1D was seen in most strata in subgroup analyses (Supplementary Table S7). The adjusted HR was higher in males (aHR = 1.75 [1.35–2.26] vs. females: aHR = 1.25 [0.85–1.84], P_*for interaction*_ < 0.05), and in individuals aged between 18 and 28 (aHR = 1.76 [1.35–2.29] vs. those younger than 18: aHR = 1.31 [0.91–1.88], P_*for interaction*_ < 0.05). The elevated HR was driven by UC, in which similar strata patterns were observed (Supplementary Table S8). We also observed a higher hazard of developing T1D in IBD patients with PSC (aHR = 13.02 [2.32–73.05]), although the number of events was low (six in IBD patients and three in reference individuals, Supplementary Table S9).

An increased hazard of developing T1D was also observed in patients with IBD and UC across all sensitivity analyses, including analyses that were restricted to patients naïve to IBD surgery, steroids, or biologics (Supplementary Table S10). The associations were robust but attenuated when we discarded the first one or three years of follow-up or applied stricter definitions for T1D (Supplementary Table S10).

In the sibling comparison analyses, we identified 14,511 IBD patients (CD: 5250; UC: 7174; IBD-U: 2087) and 22,201 eligible IBD-free full siblings (Supplementary Table S11). Compared with their IBD-free siblings, patients with IBD were older and had a higher prevalence of other autoimmune diseases before IBD diagnosis. Consistent with the findings from the reference individual comparison analyses, the hazard of T1D was higher in patients with IBD than their siblings (aHR = 1.44 [0.97–2.15]) and was driven by UC (aHR = 1.90 [1.12–3.24]) (Fig. 2 & Supplementary Table S12).

### Baseline characteristics of the case–control study

We enrolled 87,001 IBD cases and 431,054 matched controls in the case–control study. IBD patients were diagnosed at a median age of 42.2 years and 48.3% of them were female (Table 2). Prevalence of other autoimmune diseases was also higher in IBD patients (13.5% vs. 7.4% in controls). Around 1.3% of IBD patients had PSC, 14.6% of CD patients had a colonic involvement, and 15.5% of UC patients had an extensive colitis (Supplementary Table S13).

**Table 2:** Characteristics of patients with IBD and their matched controls in the case–control study, n (%).

	Controls	Patients with IBD	Subtypes of IBD		
			CD	UC	IBD-U
N	431,054	87,001	25,783	48,406	12,812
Age at index date, years^a^					
Mean ± SD	43.9 ± 19.5	43.9 ± 19.4	41.6 ± 19.6	44.5 ± 18.8	46.2 ± 20.9
Median (IQR)	42.2 (27.8–59.0)	42.2 (27.9–58.9)	39.3 (25.0–56.6)	42.8 (29.3–58.9)	45.6 (28.6–63.3)
Age at index date, years					
<18	35,326 (8.2)	7081 (8.1)	2865 (11.1)	3050 (6.3)	1166 (9.1)
18–<40	164,246 (38.1)	33,177 (38.1)	10,275 (39.9)	18,687 (38.6)	4215 (32.9)
40–<60	129,826 (30.1)	26,258 (30.2)	7298 (28.3)	15,306 (31.6)	3654 (28.5)
≥60	101,656 (23.6)	20,485 (23.6)	5345 (20.7)	11,363 (23.5)	3777 (29.5)
Female	208,390 (48.3)	42,057 (48.3)	13,422 (52.1)	22,308 (46.1)	6327 (49.4)
Born in Nordic country	384,331 (89.2)	80,180 (92.2)	23,461 (91.0)	45,037 (93.0)	11,682 (91.2)
Calendar period at index date					
1987–2001	157,592 (36.6)	31,710 (36.5)	10,075 (39.1)	18,887 (39.0)	2748 (21.5)
2002–2009	138,706 (32.2)	28,000 (32.2)	7990 (31.0)	15,915 (32.9)	4095 (32.0)
2010–2017	134,756 (31.3)	27,291 (31.4)	7718 (29.9)	13,604 (28.1)	5969 (46.6)
Educational attainment, years					
0–9	97,772 (22.7)	20,098 (23.1)	6166 (23.9)	10,845 (22.4)	3087 (24.1)
10–12	172,605 (40.0)	36,419 (41.9)	10,684 (41.4)	20,381 (42.1)	5354 (41.8)
≥13	115,492 (26.8)	22,709 (26.1)	6127 (23.8)	13,080 (27.0)	3502 (27.3)
Missing	45,185 (10.5)	7775 (8.9)	2806 (10.9)	4100 (8.5)	869 (6.8)
History of autoimmune diseases	31,679 (7.4)	11,777 (13.5)	4288 (16.6)	4998 (10.3)	2491 (19.4)

CD: Crohn’s disease; IBD(-U): inflammatory bowel disease (unclassified); IQR: interquartile range; SD: standard deviation; UC: ulcerative colitis.

a Index date: date of IBD diagnosis for patients with IBD, and date of selection for their matched controls.

### IBD and the odds of prior T1D

**Fig. 3:**
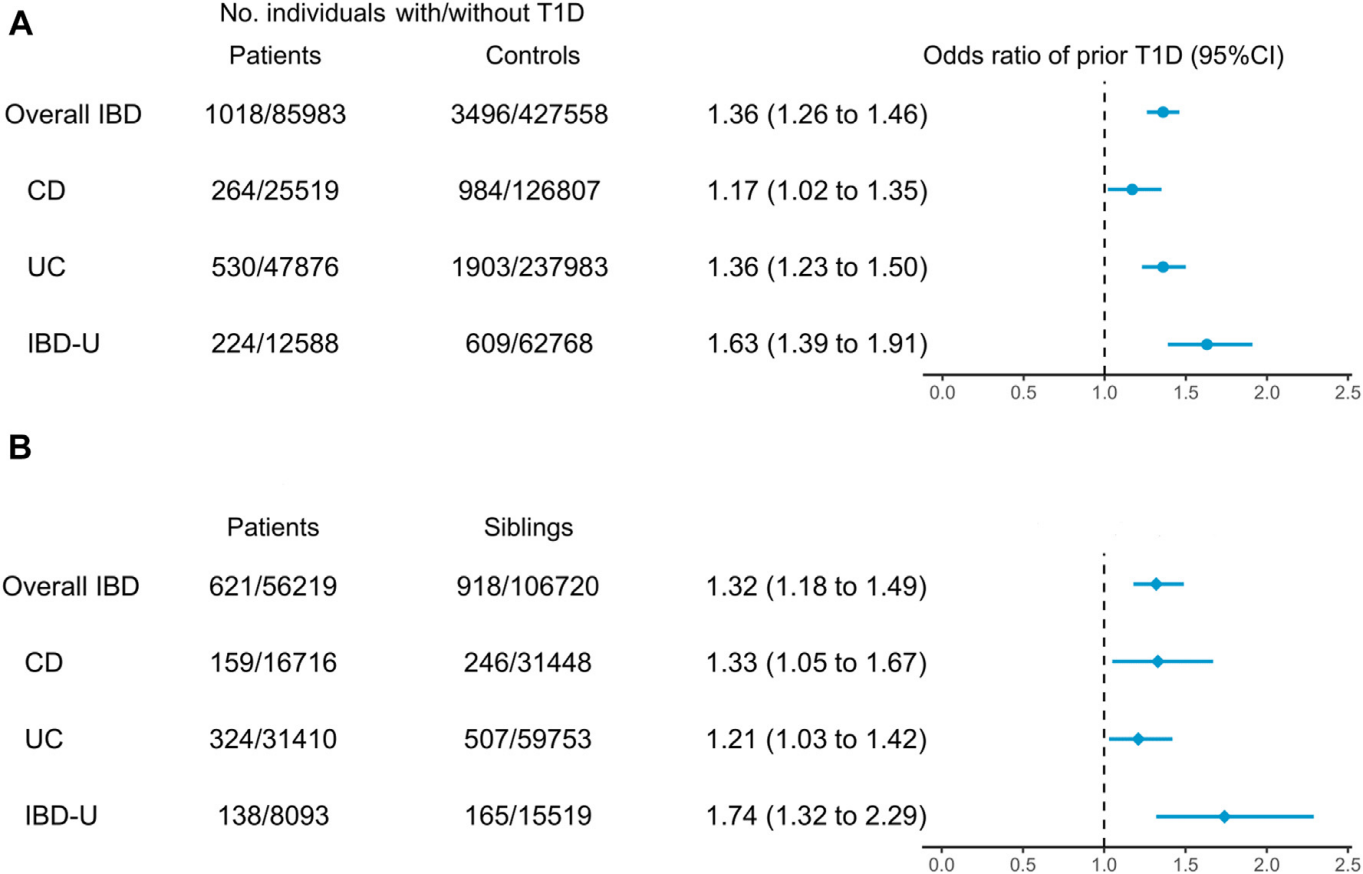
Odds ratios for prior type 1 diabetes (T1D) in patients with inflammatory bowel disease (IBD), compared with their matched reference individuals (A) and full siblings (B), with 95% confidence interval (CI). The odds ratio was estimated from the conditional logistic regression model in Model 2. CD: Crohn’s disease; IBD-U: IBD-unclassified; and UC: ulcerative colitis.

A total of 1018 (1.2%) IBD patients and 3496 (0.8%) controls had been previously diagnosed with T1D. The median (IQR) age of T1D diagnosis was 47.8 (23.1–63.4) and 40.8 (18.3–63.2), respectively. Compared with controls, patients with IBD had higher odds of having a prior diagnosis of T1D (aOR = 1.36 [1.26–1.46]). The odds of prior T1D were increased in all IBD subtypes, including CD (aOR = 1.17 [1.02–1.35]), UC (aOR = 1.36 [1.23–1.50]), and IBD-U (aOR = 1.63 [1.39–1.91]) (Fig. 3 & Supplementary Table S14).

### Subgroup and sensitivity analyses in the case–control study

A positive association between IBD and prior T1D was observed in almost all subgroups by sex, calendar period, educational attainment, and history of autoimmune diseases, with aORs increased by thirty to fifty percent (Supplementary Table S15). The association tended to be stronger in patients diagnosed with IBD older than 40 years (aOR = 1.45 [1.27–1.65] for the 40–<60 years and 1.47 [1.32–1.65] for the ≥60 years), while it was non-significant in patients diagnosed before 18 years (aOR = 1.11 [0.77–1.59]) (Supplementary Table S15). In stratified analysis by IBD phenotypes, elevated aOR of prior T1D was observed in IBD patients with PSC (aOR = 2.97 [1.83–4.81]) (Supplementary Table S16).

In sensitivity analysis, after excluding T1D identified one or three years before IBD diagnosis from the analysis, we observed an attenuated but still positive association in patients with IBD as well as UC and IBD-U (Supplementary Table S17).

In the sibling comparison analyses, prior T1D was observed in 621 IBD patients and 918 full siblings (Supplementary Table S18). Compared with their siblings, patients with IBD had higher odds of having a prior diagnosis of T1D (aOR = 1.32 [1.18–1.49]) and such positive association was irrespective of IBD subtypes (Fig. 3 & Supplementary Table S18).

## Discussion

In this nationwide matched cohort and case–control study, we found that patients with IBD (diagnosed ≤28 years) had a 58% increased hazard of developing T1D, and patients with IBD (no age restriction) had 36% higher odds of having a prior T1D. Similar associations were observed in the sibling comparison analyses, with a 44% increased hazard of developing T1D and 32% elevated odds of having a prior T1D, respectively. Our findings suggested that shared genetics (e.g., *PTPN2* and *IL2RA*^9^) and early environmental risk factors (e.g., diet^4^^,^^33^) could only partially explain the observed association between IBD and T1D. The bidirectional association was more pronounced among males, patients with UC, and those with PSC.

Until now, some large-scale studies have reported a positive association between IBD and T1D but lacked further investigation on their temporal relationships.^11^, ^12^, ^13^, ^14^ The bidirectional design of our study might account for long preclinical stages in both diseases^34^^,^^35^ and facilitate comparison with existing evidence (Supplementary Table S1). In our study, the incidence rate of T1D was 3.7 vs. 2.3 per 10,000 person-years in IBD patients and matched reference individuals, respectively. Both figures however were considerably lower than those in a Danish nationwide cohort (IBD vs. matched individuals: 21.0 vs. 7.9]),^13^ most likely because our studied population in cohort were restricted to those aged ≤28 years and therefore were much younger than that of the Danish cohort (median age: 20.8 vs. 45.8 years^13^). However, the prevalence of T1D observed in both childhood- and adult-onset IBD patients was similar to previous studies (<18 years: 0.6% vs. 0.7%^36^; ≥18 years: 0.8–2.1% vs. 1.2–1.9%^13^). The hazard of developing T1D in our cohort (aHR for IBD diagnosed 18-≤28 years: 1.76) was lower than the Danish cohort of adult IBD patients (IRR: 2.61),^13^ which could be attributed to different age distribution and adjusted covariates (see details in Supplementary Table S1). The observed aOR in our case–control study (aOR = 1.36) was comparable with most previous studies (aORs ranged from 1.4 to 1.9).^11^^,^^14^^,^^36^^,^^37^

The bidirectional association was observed only in adult-onset IBD patients, with point estimates increasing by age (as shown in the case–control study). Because the effect of genetic risk burden might be lower in T1D patients diagnosed at an older age,^38^^,^^39^ the age trend might be attributed to persistent exposure to environmental risk factors (e.g., shifted gut microbiota^7^^,^^40^) that may have emerged years before IBD diagnosis.^35^ Chronic inflammation in IBD might also accelerate the progression of β-cell dysfunction in adult-onset T1D.^38^ However, even though in our study the association of T1D with childhood-onset IBD was non-significant in either direction (aHR = 1.31 [0.91–1.88] in the cohort study; aOR = 1.11 [0.77–1.59] in the case–control study), the increased frequency of severe acute T1D complications in paediatric IBD patients justifies tailored disease management for children with both diseases.^12^ In addition, the bidirectional association was more pronounced among males, potentially suggesting that the male predominance of T1D observed in the general population^41^ might be reinforced among patients with IBD.

The stronger association of T1D with UC compared to CD has also been observed in previous studies,^13^^,^^36^^,^^37^ with several potential explanations. **First,** UC patients had a higher proportion of male sex and tended to be older than patients with CD (Table 1, Table 2). **Second,** underlying IBD phenotypes may have driven up the risk estimate especially in UC. For example, PSC that was more frequently observed in UC patients was associated with a substantially increased risk for T1D^42^ (Supplementary Table S9 and S16). The strong association between IBD-PSC and T1D was consistent with our previous findings,^43^ which could be attributed to aggravated perturbation in bile acid metabolism and interleukin-2 (IL-2) signalling,^44^ and shared genetic haplotype HLA-A1-B8-DR3.^43^ **Third,** UC is more closely related to HLA types^45^ (the main genetic susceptibility factor for T1D) and variant of some T1D risk genes has protective effect on CD (e.g., rs2476601 variant at *PTPN22*^9^).

Shared pathogenic mechanisms may explain the bidirectional association between IBD and T1D. **First,** the immunopathogenic pathways of IBD and T1D potentially crosstalk.^1^^,^^2^ Dysregulated immunity in IBD involves overactivation of antigen-presenting pathways, excessive production of proinflammatory cytokines (e.g., IL-12, interferon-γ), and loss of immune homeostasis within effector T cells,^1^^,^^2^ which potentially trigger or promote autoimmunity against pancreatic β cells.^4^ **Second,** gut microbial alterations shared between IBD and T1D may have pathogenic effects on both diseases. For instance, the loss of butyrate-producing bacteria observed in IBD and T1D has been associated with impaired epithelial function in IBD and decreased β-cell protection in T1D.^1^^,^^2^^,^^6^^,^^7^ **Third,** being both the product and the regulator in the immune-microbiota cooperation, the metabolism of bile acids may also be shifted (e.g., decreased secondary bile acid production) during early progression of IBD and T1D.^6^^,^^8^^,^^44^

The merits of our study included the matched cohort and case–control study design, the nationwide register data, and a virtually complete follow-up time. These allowed us to investigate the bidirectional association between IBD and T1D and to perform informative subgroup and sensitivity analyses after accounting for potential confounders. Our study also benefited from high PPVs for the diagnoses of IBD and T1D,^22^^,^^23^^,^^27^ which minimized the information bias commonly seen in observational studies. Moreover, the sibling comparison analyses may have helped to relieve potential intrafamilial confounders (i.e., shared genetics and early environmental factors) between IBD and T1D.^16^, ^17^, ^18^

We acknowledged the following limitations. **First,** because of the lack of primary care data and the incomplete coverage of the NPR (nationwide coverage on inpatient care from 1987 and outpatient care from 2001^20^), some cases of T1D or mild cases of IBD may not have been identified. However, the potential influence of such issues is likely small since hospital-based specialist care is typically required for both IBD and T1D.^5^^,^^46^ **Second,** due to the register-based nature of this study, we had limited information on environmental factors that may affect the risk of IBD and T1D (e.g., dietary factors^4^^,^^33^). This concern, however, was partially addressed in the sibling comparison analysis. **Third,** although we observed a positive association between IBD and T1D in patients naïve to IBD surgery, steroids, or biologics, exploring the potential influences of IBD medications and disease activity/severity on T1D was not within the scope of our study due to data unavailability. Moreover, the role of inflammatory biomarkers (e.g., faecal calprotectin and C-reactive protein) on the association was not explored. **Fourth,** due to lack of data on serological markers (e.g., glutamic acid decarboxylase antibodies), we were unable to discern different T1D endotypes. Future studies are therefore needed to further investigate the association of IBD with specific T1D endotypes such as latent autoimmune diabetes in adults.^38^ **Fifth,** although we had a large sample size in the cohort study, some analyses for CD and IBD-U and for IBD phenotypes might suffer from the inadequate statistical power. Pooling study samples across countries might be one way to circumvent this limitation in the future. **Sixth,** our study was restricted to Sweden and the Swedish healthcare system provides universal access practically free of charge. Given the differences in the incidence and prevalence of IBD and T1D, as well as the different strengths of the IBD-T1D association across countries and regions (Supplementary Table S1), caution is needed when generalizing our findings to other settings. **Finally,** due to the observational nature of this study, we cannot claim any causal relationship between IBD and T1D.

The co-occurrence of IBD and T1D imposes substantial challenges for disease management. Compared with those affected by IBD or T1D only, patients with both diseases had more frequent T1D-related complications and IBD-related hospitalizations.^12^^,^^47^ However, IBD and T1D have not been addressed as comorbidities in current management guidelines.^42^^,^^48^^,^^49^ Although the low increase in the absolute risk speaks against routine screening for T1D in IBD patients or *vice versa*, awareness of the increased co-occurrence risk may facilitate early detection of both conditions,^35^^,^^38^ especially in high-risk population such as male UC patients with PSC. Otherwise, nonspecific symptoms, such as weight loss and fatigue, might be misattributed to the initial disease. This awareness is crucial for shortening diagnostic delay and reducing the risk of disease progression and development of complications. Moreover, physicians should actively consider measuring blood glucose or HbA1c in IBD patients with unexplained weight loss or fatigue to check for diabetes. If diabetes is detected, then a β-cell autoimmunity test might be considered to determine the type of diabetes.

In conclusion, patients with IBD had a modestly increased risk of T1D both before and after IBD diagnosis, particularly in adult-onset IBD patients, males, patients with UC, and those with PSC. The bidirectional association may be partially independent of shared genetics and early environmental factors.

## Contributors

Study concept and design: JS and JFL. Acquisition of data: JFL. Drafting of the manuscript: JY, JS and JFL. Interpretation of data, and critical revision of the manuscript for important intellectual content: JS, JY, OO, JH, DB, FE, SC, JL, and JFL. Statistical analysis: JS. Funding acquisition: JS and JFL. Administrative, technical, or material support: JFL. Guarantors: JS and JFL have directly accessed and verified the underlying data reported in the manuscript and take responsibility for the integrity of the data, the accuracy of the data analysis, and the decision to submit the manuscript.

## Data sharing statement

The data set cannot be shared directly under current legislation for data protection and must be requested directly from the respective registry holders, Statistics Sweden (information@scb.se) and the Swedish National Board of Health and Welfare (registerservice@socialstyrelsen.se), after approval by the Swedish Ethical Review Authority.

## Declaration of interests

All authors have completed the ICMJE uniform disclosure form and declare: OO has been PI on projects at Karolinska Institutet financed by grants from Janssen, Pfizer, AbbVie, Takeda, Galapagos/Alfasigma, Ferring, and Bristol Myer Squibb. JH served as speaker and/or advisory board member for AbbVie, BMS, Celltrion, Eli Lilly, Ferring, Galapagos, Gilead, Index Pharma, Janssen, Medtronic, Merck, MSD, Novartis, Pfizer, Sandoz, Shire, Takeda, Thermo Fisher Scientific, Tillotts Pharma, and Vifor Pharma and received grant support from Takeda, Janssen, and MSD. FE has served as an advisory board member for Boehringer Ingelheim. JFL has coordinated a study on behalf of the Swedish IBD quality register (SWIBREG). That study received funding from Janssen Corporation. JFL has also received financial support from MSD developing a paper reviewing national healthcare registers in China. JFL has a research collaboration on celiac disease with Takeda. The other authors report no disclosures relevant to the manuscript.
