# Supplementary material for: Bidirectional association between inflammatory bowel disease and type 1 diabetes: a nationwide matched cohort and case-control study
Source: Lancet Reg Health Eur. 2024 Aug 31;46:101056. doi: 10.1016/j.lanepe.2024.101056 (PMC11402305; doi:10.1016/j.lanepe.2024.101056)
Supplement: Supplementary Tables S1–S18 [file mmc1.doc]

**Supplementary material**

**Bidirectional association between inflammatory bowel disease and type 1 diabetes: a nationwide matched cohort and case-control study**

Sun et al.

Table of Contents

[Table S1 Meta-analyses and population-based studies on the association between IBD and T1D published since 2010 4](#__RefHeading___Toc165905138)

[Table S2 ICD codes and SNOMED codes defining IBD 9](#__RefHeading___Toc165905139)

[Table S3 ICD codes for each phenotype of CD, UC, and IBD-U 10](#__RefHeading___Toc165905140)

[Table S4a Definitions of exposure, outcomes, exclusion criteria, and comorbidities 11](#__RefHeading___Toc165905141)

[Table S4b Procedural codes for IBD-related surgery 13](#__RefHeading___Toc165905142)

[Table S4c ATC codes of biologics and steroids for IBD 15](#__RefHeading___Toc165905143)

[Table S5 Clinical phenotypes of patients with IBD in the cohort study, n (%) 16](#__RefHeading___Toc165905144)

[Table S6 Incident T1D in patients with IBD and their matched reference individuals in the cohort study 17](#__RefHeading___Toc165905145)

[Table S7 Subgroup analyses of incident T1D in patients with IBD and their matched reference individuals in the cohort study 18](#__RefHeading___Toc165905146)

[Table S8 Subgroup analyses of incident T1D in patients with CD, UC, IBD-U and their matched reference individuals in the cohort study 19](#__RefHeading___Toc165905147)

[Table S9 Incident T1D in patients with IBD and their matched reference individuals in the cohort study, by phenotypes according to the Montreal Classification 20](#__RefHeading___Toc165905148)

[Table S10 Sensitivity analyses of incident T1D in patients with IBD and their matched reference individuals in the cohort study 21](#__RefHeading___Toc165905149)

[Table S11 Characteristics of patients with IBD and their IBD-free full siblings in the cohort study 23](#__RefHeading___Toc165905150)

[Table S12 Incident T1D in patients with IBD and their IBD-free full siblings in the cohort study 24](#__RefHeading___Toc165905151)

[Table S13 Clinical phenotypes of patients with IBD in the case-control study, n (%) 25](#__RefHeading___Toc165905152)

[Table S14 Prior T1D in patients with IBD in the case-control study 26](#__RefHeading___Toc165905153)

[Table S15 Subgroup analyses of prior T1D in patients with IBD in the case-control study 27](#__RefHeading___Toc165905154)

[Table S16 Subgroup analyses of prior T1D in patients with IBD in the case-control study, by phenotypes according to the Montreal Classification 28](#__RefHeading___Toc165905155)

[Table S17 Sensitivity analyses of prior T1D in patients with IBD in the case-control study 29](#__RefHeading___Toc165905156)

[Table S18 Prior T1D in patients with IBD and their IBD-free full siblings in the case-control study 30](#__RefHeading___Toc165905157)

Table S1 Meta-analyses and population-based studies on the association between IBD and T1D published since 2010

| **Meta-analysis** | | | | | | | | | |
| --- | --- | --- | --- | --- | --- | --- | --- | --- | --- |
| **First author, publication year,**  **place** | | **Updated until** | | **No. of studies** | **I^2^, %** | | **Pooled estimates (95% CI)** | | |
| Lu^1^, 2020, China | | 31 December 2019 | | 6 | CD: 70; UC: 80 | | OR in CD: 1.16 (95%CI: 0.87-1.55)  UC: 1.20 (0.90-1.59) | | |
| **Individual studies** | | | | | | | | | |
| **First author, publication year,  place,  data source, study period, follow-up time, study type** | **Sample size** | **Age at IBD diagnosis, years** | **Female, %** | **Number of T1D in IBD** | **Incidence rate of outcomes, per 10,000 person years** | **First year of follow-up was included or not** | | **Covariates** | **Relevant findings** |
| **Current study** | | | | | | | | | |
| Sun,  Sweden, Nationwide,  1969 through 2021 (cases identified from 1987 to 2017),  Median 14.6 (IQR: 9.2-20.8) years,  Cohort and matched case-control study | Cohort: IBD: 20,314; **CD: 7,277; UC: 10,112; IBD-U: 2,925**  Case-control study: IBD: 87,001; CD:25,783;UC: 48,406; IBD-U: 12,812 | Cohort: Median 20.8 (IQR: 16.5-24.5) years,  age restricted ≤28 years  Case-control study: Median 42.2 (IQR: 27.9-58.9) years, no age restriction | Cohort: 46.4;  Case-control study:  48.3 | Cohort:  IBD: 116;  CD: 33;  UC: 68;  IBD-U: 15  Case-control study:  IBD: 1,018; CD: 264; UC: 530; IBD-U: 224 | Cohort: IBD: 3.7 vs 2.3;  CD: 2.9 vs 2.5;  UC: 4.2 vs 2.2;  IBD-U: 3.9 vs 2.4 | Included, first one or three follow-up years excluded in sensitivity analysis | | (Model 1)  1. birth year  2. sex  3. county of residence  4. calendar year  (Model 2)  5. country of birth  6. educational attainment  7. autoimmune diseases (other than IBD and T1D) | Cohort: the aHR of incident T1D was increased in IBD (aHR=1.58, 95% CI: 1.27-1.95) and UC (aHR=2.02, 1.51-2.70), but not in CD (aHR=1.11, 0.76-1.64) or IBD-U (aHR=1.48, 0.82-2.69)  Case-control study: the aOR of T1D exposure was higher in IBD patients (aOR=1.36, 1.26-1.46) as well as in all IBD subtypes [aOR: (1.17,1.02-1.35) for CD; (1.36, 1.23-1.50) for UC; and (1.63; 1.39-1.91) for IBD-U] |
| **Individual studies since 2010 (*: also included in abovementioned meta-analysis)** | | | | | | | | | |
| Conrad^2^, 2023, UK, Nationwide primary and secondary care register,  1 January 1985 to 30 June 2019, Mean: 6.17 years for the whole cohort, cohort | all: 978,872; sample for specific autoimmune disease not reported | All patients with incident autoimmune disease, Mean (SD): 54.0 (21.4) | All patients with incident autoimmune disease: 63.9 | Not reported for specific population with certain autoimmune disease. Incident IBD overall: 66,173 Incident T1D overall: 9,022 | Not reported | Included | | Age and sex standardized with European general population | (In supplementary) The age- and sex- standardized IRR of developing IBD in T1D is 1.8 (95% CI: 1.3-2.4); IRR of developing T1D in IBD is 1.9 (1.0-3.4). |
| Jasser-Nitsche^3^, 2021,  Multicenter biennial survey in Germany and Austria,  January 1995 to March 2015, cross-sectional study | T1D: 65,147 | All patients ≤18 years,  T1D only:  Mean (SD): 8.59 (4.36);  T1D+IBD: 10.77 (4.32) | T1D only: 47.3;  T1D+IBD:  49.2 | T1D+IBD: 63 (0.097%)  T1D+CD: 26 (0.040%), T1D+UC: 33 (0.050%), T1D+IBD-U: 4 (0.006%) | NA | NA | | RR:  prevalence ratio standardized by age;  Occurrence of complications:  Poisson regression model adjusted for  1. age  2. sex  3. T1D durations | The age-standardized prevalence of IBD (for CD and UC but not for IBD-U) in T1D was significantly higher than that in the general population.  RR was 3.25 (95% CI: 2.17-4.88) for IBD-overall, 2.06 (1.17-3.65) for CD, and 4.67 (2.35-9.26) for UC. Comorbid IBD was associated with a lower BMI and a higher prevalence of steroids use. Severe hypoglycemia (but not for other acute complications such as hypoglycemia with coma and diabetic ketoacidosis) was also seen more frequently in T1D patients with comorbid IBD (0.33±0.22 vs 0.16±0.01 events per person year). |
| Gherisin^4^*, 2019, Israel, Jewish population-based annual health evaluation during 2004 (2002?) to 2016, cross-sectional study | IBD: 891; CD: 595 (67%); UC: 296 (33%) | All IBD patients≤18 years, Median (IQR): 15 (12.5-17) | IBD: 36 | 0 in both CD and UC | NA | NA | | NA | T1D was not more common in either CD or UC. |
| Park^5^, 2019, Korea, Nationwide administrative register, January 2012 to December 2013 (followed to 2016), Mean: 4.57 years,  cohort | IBD: 35,581; CD: 11,803; UC: 23,737; | Mean (SD): IBD: 41.7 (16.4); CD: 32.3 (14.0); UC: 46.4 (15.4) | IBD: 38.4; CD: 29.1; UC: 43.1 | IBD: 49; CD: 12; UC: 37 | IBD: 3.01; CD: 2.21; UC: 3.41 | Included | | Only adjusted with matching variables  1. age  2. sex | No significantly increased hazard of developing T1D in patients with IBD (aHR: 0.99, 95% CI: 0.72-1.35), CD (1.54, 0.80-2.99), or UC (0.88, 0.62-1.26). |
| Burisch^6^, 2019, Denmark, Nationwide in-and outpatient register, 1 January 2007 to 31 December 2016, Median (IQR) follow-up for immune-mediated inflammatory diseases overall: 5.4 years (2.9-7.8), cohort | IBD: 14,377; CD: 3,879 (27%); UC: 9,212 (64%); IBD-U: 1,286 (9%) | Overall cohort: Median (IQR): IBD: 45.8 (33.8-60.7); CD: 43.9 (31.9-58.7);  UC: 48.8 (34.8-61.9); IBD-U: 45.2 (31.7-56.9) IBD without history of any immune-mediated inflammatory diseases: 11,777 | Overall cohort: IBD: 52.3; CD: 55.9;  UC: 50.5; IBD-U: 54.8 | Occurrence before the diagnosis of IBD: CD: 72; UC: 175; IBD-U: 16;  Occurrence after the diagnosis of IBD:  CD: 26;  UC: 109;  IBD-U: 27; | IBD: 21.0 (95%CI: 18.0-24.4) vs 7.9 (7.1-8.9); CD: 13.2 (9.0-19.4) vs 6.2 (4.9-8.0); UC: 22.8 (18.9-27.5) vs 8.5 (7.4-9.8); IBD-U: 27.5 (18.9-40.1) vs 8.5 (6.3-11.6) | Included | | 1. sex  2. date-of-birth  (+) covariates in Poisson regression model:  3. socioeconomic status  4. medications for IBD (time-dependent variables) | (In supplementary)  Higher incidence of T1D was observed in IBD (IRR: 2.61, 95% CI: 2.17-3.17), including CD (2.11, 1.33-3.33), UC (2.65, 2.10-3.34), and IBD-U (3.22, 1.99-5.22). IBD patients who were treated with infliximab were at reduced risk of developing IMIDs (CD (aOR: 0.52, 95% CI: 0.34–0.81) and UC (0.47, 0.29–0.76)). Risk of surgery increased in comorbid CD and immune-mediated inflammatory diseases (after CD onset (2.30, 1.46–4.20) but not in comorbid UC and immune-mediated inflammatory diseases. |
| Bar Yehuda^7^*, 2019, Israel,  Population-based administrative register, (for incident cohort): 2005-unknown, Median (IQR): IBD: 5.41 years (2.93-8.13); CD: 5.16 years (2.78-8.0); UC: 5.62 years (3.09-8.35), cohort | Prevalence cohort:  IBD: 12,625; CD: 6,364; UC: 6,261  Incidence cohort:  IBD: 5,298;  CD: 2,849;  UC: 2,449 | Mean (SD) in prevalence cohort:  IBD: 47.8 (18.6); CD: 43.7 (17.6); UC: 51.9 (18.7)  Mean (SD) in incidence cohort:  IBD: 42.4 (17.8);  CD: 37.6 (17.4);  UC: 45.2 (17.6) | Prevalence cohort:  IBD: 49.1;  CD: 47.7; UC: 51.0  Incidence cohort:  IBD: 48.9;  CD: 48.5;  UC: 49.5 | Prevalence cohort of IBD: (95+169)/12,625 (2.1%); CD: 95/6,364 (1.5%); UC: 169/6,261 (2.7%)  Incidence cohort of IBD medication users: Anti-TNF-alpha users: 12/955 (1.3%); Non-anti-TNF-alpha users:70/4,343 (1.6%); Thiopurine users: 26/1,813 (1.4%); Non-thiopurine users: 56/3,485 (1.6%) | Not reported | Included | | Matching variables:  1. age  2. sex  3. socio-economic status  (+) covariates for multivariate logistic regression model:  4. IBD subtype  5. follow-up duration | There were higher odds of concomitant T1D in IBD compared with controls (OR: 1.41, 95% CI: 1.15-1.71). The odds of T1D in UC were higher than CD (OR: 1.83, 1.41-2.38). None of anti-TNF-alpha, thiopurine, or 5-aminosalicylates was associated with increased prevalence of T1D in IBD. |
| Halling^8^*, 2017, Denmark, Nationwide in- and outpatient register,  Data retrieved on 16 December 2013, cross-sectional study | IBD: 47,325; CD: 13,343;  UC: 31,066; CD+UC: 2,916 | Mean: IBD: 42; CD: 37;  UC: 44; CD+UC: 34 | IBD: 54; CD: 58;  UC: 53; CD+UC: 56 | IBD: 1,682; CD: 359;  UC: 1,002; CD+UC: 103 | NA | NA | | Only adjusted with variables matching:  1. age;  2. sex;  3. municipality | Increased odds of concomitant T1D in IBD. The OR for IBD overall was 1.7 (95% CI: 1.6-1.9), for CD was 1.7 (1.4-1.9), for UC was 1.7 (1.6-1.8), and for CD+UC was 2.9 (2.2-3.9). |
| Virta^9^*, 2012, Finland,  Nationwide register,  January 1, 1994 to December 31, 2008, case-control study | IBD: 595; CD: 233;  UC: 362 | Median (IQR): IBD: 10.2 (6.1-12.6); CD: 10.9 (7.7-13.0);  UC: 9.5 (5.4-12.2) | IBD: 43; CD: 37; UC: 48 | IBD: 2; CD: 1; UC: 1 | NA | NA | | Matching variables:  1. age;  2. sex;  3.place of residence at birth | Non-significant association between IBD (including CD and UC) and T1D |
| Kappelman^10^, 2011, the US,  Population-based administrative register,  January 1, 2003 to December 31, 2004, cross-sectional study | IBD: 1,242; CD: 737; UC: 488; | Mean (SD), Median: IBD: 15.0 (3.4), 16; CD: 15.0 (3.2), 16; UC: 14.8 (3.7), 16 | IBD: 45; CD: 44; UC: 47 | IBD: 15; CD: 5; UC: 10 | NA | NA | | Matching variables:  1. age;  2. sex;  3. region; | Higher odds of T1D was observed in IBD overall (OR: 1.9, 95% CI: 1.0-3.8) and for UC (OR: 2.7, 1.1-6.6), but not for CD (1.4, 0.5-4.0). Sensitivity analysis excluding patients on anti-TNF-alpha (to avoid misclassification for reimbursement purposes) confirmed the main finding. |

aHR: adjusted hazard ratio; CD: Crohn’s disease; CI: confidence interval; CKD: chronic kidney diseases; COPD: chronic obstructive pulmonary disease; IBD(-U): inflammatory bowel disease (unclassified); IMID: immune-mediated inflammatory disease; IRR: incidence rate ratio; IQR: interquartile range; NA: not applicable; OR: odds ratio; SD: standard deviation; T1D: type 1 diabetes; UC: ulcerative colitis.

Table S2 ICD codes and SNOMED codes defining IBD

| **IBD subtypes** ^a^ | **ICD-7  (1964-1968)** | **ICD-8  (1969-1986)** | **ICD-9  (1987-1996)** | **ICD-10  (1997-)** | **SNOMED codes ^b^** |
| --- | --- | --- | --- | --- | --- |
| UC | 572,20; 572,21; 578,03 | 563,1; 563,10; 569,02; 569,04 | 556 | K51 | D6255 or M41; M42; M43; M44; M463; or M47 |
| CD | 572,00; 572,09 | 563,00 | 555 | K50 | D6216 or M41; M42; M43; M44; M463; or M47 |
| IBD-U | UC + CD | UC + CD or 563; 563,0; 563,9; 563,98; 563,99 | UC + CD | UC + CD or K523 | D6214 or M41; M42; M43; M44; M463; or M47 |
| CD: Crohn’s disease; IBD(-U): inflammatory bowel disease (unclassified); ICD: International Classification of Disease; SNOMED: Systematized Nomenclature of Medicine; UC: ulcerative colitis.  ^a^ Diagnosis criteria^11^: ≥1 ICD code for IBD **AND** ≥1 SNOMED code for IBD (positive predictive value: 95%^12^)  ^b^ In SNOMED codes, D codes are the diagnostic codes, D6255 for example was the diagnostic code for UC. Meanwhile, codes starting with “M” (one unspecific SNOMED code) refer to all codes starting with the respective code.  Subtypes of IBD were defined according to the first ICD and SNOMED codes only (i.e., no information after start of follow-up contributed to the subtype definition); in case one individual had one ICD code for IBD and one unspecific SNOMED code (i.e., the "M" code), the IBD subtype was solely determined by the ICD code. | | | | | |

Table S3 ICD codes for each phenotype of CD, UC, and IBD-U

| **Montreal classification** | **Diagnostic codes** |
| --- | --- |
| CD location ^a^ |  |
| Ileal (L1) | K500 |
| Colonic (L2) | K501 |
| Ileocolonic or location not defined (L3/LX) | K508; K509 |
| Perianal disease modifier | Any of the diagnostic codes: K603; K604; K605; K610; K611; K612; K613;K614; K624 OR any of the surgical procedure codes: JHD20; JHD30; JHD33; JHD50; JHD60; JHD63; JHA00; JHA20; JHW96 |
| UC extent ^a^ |  |
| Proctitis (E1) | K512 |
| Left-sided colitis (E2) | K513; K515 |
| Extensive colitis (E3) | K510 |
| Extent not defined (EX) | K514; K518; K519 |
| Primary sclerosing cholangitis ^b^ | ICD-9 (1987-1996): 576B |
|  | ICD-10 (1997-): K830 |
| Other extraintestinal manifestations | ICD-9: 695C; 364; 713B; 720A; 720C; 720W; 720X |
|  | ICD-10: L52; L88; L982; H20; M074; M075; M076; |
|  | M091; M092; M45; M460; M461; M468; M469; M139; M255 |

CD: Crohn’s disease; E: extent; IBD(-U): inflammatory bowel disease (unclassified); ICD: International Classification of Disease; L: location; UC: ulcerative colitis.

^a^ The Montreal classification to validate and to define disease phenotypes was available since the use of ICD-10 in Sweden (1997-). All codes are captured in the Swedish National Patient Register (prospectively recorded in routine clinical practice).

In this study, we categorized Crohn's disease location into two groups: ileal (L1)/ileocolonic (L3)/unknown (LX) or colonic (L2), and categorized ulcerative colitis extent into three groups: proctitis (E1)/left-sided colitis (E2), extensive colitis (E3) or extent not defined (EX).

^b^ We restricted our use to ICD-9 and ICD-10 codes since we believe that earlier ICD codes for extraintestinal inflammation are less reliable, particularly for primary sclerosing cholangitis. The validity of the PSC codes has not formally been tested in Sweden^13^.

**Table S4a Definitions of exposure, outcomes, exclusion criteria, and comorbidities**

| **Exposure and outcome** | **ICD codes** |
| --- | --- |
| T1D | ICD-8: 250 + individuals aged ≤ 30 years |
|  | ICD-9: 250 + individuals aged ≤ 30 years; |
|  | ICD-10: E10 |
| IBD | See Table S2 |
| **Exclusion criteria** | **ICD codes** |
| Gestational diabetes |  |
|  | ICD-10: O24 |
| Additional excluded conditions in sensitivity analysis: |  |
| Diabetes insipidus and nephrogenic diabetes |  |
|  | ICD-8: 253; 273,81 |
|  | ICD-9: 253F, 588B |
|  | ICD-10: E232; N251 |
| Pancreatitis, acute or chronic |  |
|  | ICD-8: 577 |
|  | ICD-9: 577 |
|  | ICD-10: K85; K86 |
| Pancreatic insufficiency | ATC: A09AA02 |
| Pancreatic cancer |  |
|  | ICD-8: 157 |
|  | ICD-9: 157 |
|  | ICD-10: C25 |
| **Comorbidities** | **ICD codes** |
| Autoimmune diseases other than IBD | ICD-8: 135; 136,03; 136,07; 242,00; 242,09; 242,10; 242,2; 244; 245; 246; 255,1; 269,0; 287,0; 323,00; 340; 341,01; 354; 355; 390; 391; 392; 443,0; 443,1; 446,0; 446,1; 446,2; 446,30; 446,38; 446,9; 447; 540-543; 571,9; 693,99; 694,00; 694,02; 696; 709,05; 711; 712; 713,1; 714; 715; 716,0; 716,1; 717; 726; 733,00; 734,0; 734,1; 734,90; 734,91; 734,98; 734,99 |
|  | ICD-9: 099D; 135; 136B; 242A; 242B; 242C; 242D; 242X; 244; 245; 255E; 287A; 323G; 340; 341A; 357A; 358A; 390-392; 443A; 443B; 446A; 446B; 446C; 446E; 446E; 446F; 446H; 447G; 540-543; 571G; 579A; 694A; 694C; 694E; 694F; 694G; 696; 709A; 710A; 710B; 710D; 710E; 710I; 714; 720; 725 |
|  | ICD-10: D690; D86; E034; E035; E038; E039; E050; E051; E052; E055; E058; E059; E061; E063; E069; E271; E272; G040; G048; G35; G360; G610; G611; G618; G619; G700; I00; I010; I011; I012; I018; I019; I020; I029; I730; I731; K35-K37; K743; K900; L100; L12; L130; L40; L80; M02; M050; M051; M052; M053; M058; M059; M060; M061; M062; M063; M064; M068; M069; M080; M081; M082; M083; M084; M088; M089; M090; M091; M092; M098; M300-M303; M310; M313-M317; M32; M330-M332; M339; M34; M350-M353; M45 |

ATC: Anatomical Therapeutic Chemical; IBD: inflammatory bowel disease; ICD: international classification of diseases; T1D: type 1 diabetes

**Table S4b** Procedural codes for IBD-related surgery

| **Classification of surgical procedures** | **5/6th edition (1963-1996)** | **7th edition (1997-)** | |
| --- | --- | --- | --- |
| Colectomy |  |  | |
| 1) Subtotal colectomy with end ileostomy |  |  | |
| Colectomy and ileostomy with closure of the rectum | 4651 | JFH10 | |
| Laparoscopic colectomy and ileostomy |  | JFH11 | |
| Other colectomy |  | JFH96 | |
| 2) Colectomy with ileorectal anastomosis |  |  | |
| Colectomy with ileorectal anastomosis | 4650 | JFH00 | |
| Laparoscopic colectomy with ileorectal anastomosis |  | JFH01 | |
| Ileorectal anastomosis |  | JFC40 | |
| Laparoscopic ileorectal anastomosis |  | JFC41 | |
| Closure of enterostomy with anastomosis to the rectum |  | JFG29 | |
| Closure of enterostomy with anastomosis to the colon |  | JFG26 | |
| 3) Partial colectomies |  |  | |
| Right-sided colectomy | 4641 | JFB30, JFB31 | |
| Resection of the colon transversum | 4643 | JGB40, JFB41 | |
| Left-sided colectomy | 4640 | JFB43, JFB44 | |
| Resection of the sigmoid colon | 4644 | JFB46, JFB47 | |
| Other colon resection | 4649 | JFB50, JFB51 | |
| 4) Proctocolectomy with ileal pouch-anal anastomosis |  |  | |
| Colectomy, rectal mucosectomy and ileoanal anastomosis without ileostomy |  | JFH30 | |
| Colectomy, rectal mucosectomy and ileoanal anastomosis and ileostomy |  | JFH33 | |
| Mucosectomy and ileoanal anastomosis after previous colectomy | 4654 | JGB50 | |
| Extirpation of rectum or making of an ileoanal anastomosis after previous colectomy |  | JGB60 | |
| 5) Continent ileostomy at time of colectomy |  |  | |
| Proctocolectomy with continent ileostomy, “Kock” | 4653 | JFH40 | |
| Converting a conventional ileostomy to a continent ileostomy |  | JFG60 | |
| 6) Proctocolectomy |  |  | |
| Proctocolectomy with ileostomy | 4652 | JFH20 | |
| Other bowel surgery |  |  | |
| Strictureplasty to the small bowel |  | JFA60 | |
| Strictureplasty to the colon |  | JFA63 | |
| Closure of small intestinal fistula |  | JFA76 | |
| Closure of colonic fistula |  | JFA86 | |
| Colonic and/or small bowel resection | 4630, 4631, 4640-4649 | JFB | |
| Formation of stoma |  | JFF | |
| Operations on intestinal stoma or reservoir |  | JFG | |
| Other operation of the small bowel and/or colon | 4660-4668, 4700-4739, 4790-4798 | JFW96 | |
| Other laparoscopic operation of the small bowel and/or colon |  | JFW97 | |
| Rectal resection | 4820-4828 | JGB | |
| Perianal surgery |  |  | |
| Perianal incision and drainage | 4900 | JHA00 | |
| Dilatation of the anal sphincter | 4960 | JHD00 | |
| Lay open or excision of perianal fistula | 4920, 4922-4924 | JHD20 | |
| Partial lay open or excision of perianal fistula (including seton placement) | 4970-4971 | JHD30 | |
| Completion lay open or excision of perianal fistula |  | JHD33 | |
| Excision of perianal fistula with advancement flap |  | JHD50 | |
| Occlusion of perianal fistula with collagen plug |  | JHD60 | |
| Occlusion of perianal fistula with fibrin glue |  | JHD63 | |
| Other anal or perianal surgery (e.g., examination under anesthesia) | 4999 | JHW96 | |
| IBD: inflammatory bowel disease. |  | |  |

**Table S4c** ATC codes of biologics and steroids for IBD

| **Medications** | **ATC code** |
| --- | --- |
| Steroids |  |
| Systemic corticosteroids |  |
| Betamethasone | H02AB01 |
| Dexamethasone | H02AB02 |
| Methylprednisolone | H02AB04 |
| Prednisolone | H02AB06 |
| Prednisone | H02AB07 |
| Hydrocortisone | H02AB09 |
| Cortisone | H02AB10 |
| Corticosteroids acting locally |  |
| Hydrocortisone | A07EA02 |
| Budesonide | A07EA06 |
| Biologics |  |
| Anti-TNF treatment |  |
| Infliximab | L04AB02 (L04AA12 before 2008) |
| Adalimumab | L04AB04 (L04AA17 before 2008) |
| Golimumab | L04AB06 |
| Other biologics/tofacitinib |  |
| Vedolizumab | L04AA33 |
| Tofacitinib | L04AA29 |
| Ustekinumab | L04AC05 |
| ATC: Anatomical Therapeutic Chemical; IBD: inflammatory bowel disease; TNF: tumour necrosis factor | |

**Table S5 Clinical phenotypes of patients with IBD in the cohort study, n (%)**

|  | | |  | **IBD** | **Subtypes of IBD** | | |
| --- | --- | --- | --- | --- | --- | --- | --- |
|  | | |  |  | **CD** | **UC** | **IBD-U** |
| Extraintestinal manifestations at index date | | |  |  |  |  |  |
| PSC | | |  | 276 (1.4) | 38 (0.5) | 193 (1.9) | 45 (1.5) |
| Other extraintestinal manifestations | | |  | 663 (3.3) | 297 (4.1) | 238 (2.4) | 128 (4.4) |
| Montreal Classification CD at index date | | |  |  |  |  |  |
| No location information | | |  |  | 1296 (17.8) | - | - |
| L1, L3/LX (Ileal, ileocolonic or location not defined) | | |  |  | 4953 (68.1) | - | - |
| L2 (Colonic) | | |  |  | 1028 (14.1) | - | - |
| Perianal | | |  |  | 516 (7.1) | - | - |
| Montreal Classification UC at index date | | |  |  |  |  |  |
| No extent information ^a^ | | |  |  | - | 1819 (18.0) | - |
| E1/E2 (Proctitis, left-sided colitis) | | |  |  | - | 2387 (23.6) | - |
| E3 (Extensive colitis) | | |  |  | - | 2042 (20.2) | - |
| EX (Extent not defined) | | |  |  | - | 3864 (38.2) | - |
|  |  | CD: Crohn’s disease; E: Extent; IBD-U: inflammatory bowel disease unclassified; L: location; PSC: primary sclerosing cholangitis; UC: ulcerative colitis. | | | | | |
|  |  | ^a^ No extent information refers to patients who were diagnosed with UC before the introduction of ICD-10 in 1997. | | | | | |

Table S6 Incident T1D in patients with IBD and their matched reference individuals in the cohort study

|  | No. of events (IR, per 10,000 person-years) | | IR difference (95%CI), per 10,000 person-years | HR (95%CI) | |
| --- | --- | --- | --- | --- | --- |
|  | Patients | References |  | Model 1 ^a^ | Model 2 ^b^ |
| Overall IBD | 116 (3.7) | 353 (2.3) | 1.4 (0.6 to 2.1) | 1.62 (1.31 to 2.00) | 1.58 (1.27 to 1.95) |
| CD | 33 (2.9) | 139 (2.5) | 0.4 (-0.7 to 1.4) | 1.14 (0.78 to 1.67) | 1.11 (0.76 to 1.64) |
| UC | 68 (4.2) | 169 (2.2) | 2.0 (1.0 to 3.1) | 2.03 (1.53 to 2.70) | 2.02 (1.51 to 2.70) |
| IBD-U | 15 (3.9) | 45 (2.4) | 1.5 (-0.6 to 3.6) | 1.59 (0.89 to 2.87) | 1.48 (0.82 to 2.69) |
| CD: Crohn's disease; CI: confidence interval; HR: hazard ratio; IBD(-U): inflammatory bowel disease (unclassified); IR: incidence rate; T1D: type 1 diabetes; UC: ulcerative colitis. | | | | | |
| ^a^ Conditioned on the matching variables (birth year, sex, county of residence, and calendar year). | | | | | |
| ^b^ Further adjusted for country of birth, educational attainment, and autoimmune diseases. | | | | | |

**Table S7 Subgroup analyses of incident T1D in patients with IBD and their matched reference individuals in the cohort study**

| **Group** | **T1D** | | | |
| --- | --- | --- | --- | --- |
|  | **No. of events (IR, per 10,000 person-years)** | | **IR difference (95%CI), per 10,000 person-years** | **HR (95%CI) ^a^** |
|  | **Patients** | **References** |  |  |
| Sex |  |  |  |  |
| Male | 82 (4.9) | 226 (2.8) | 2.1 (1.0 to 3.2) | 1.75 (1.35 to 2.26) |
| Female | 34 (2.3) | 127 (1.8) | 0.5 (-0.3 to 1.3) | 1.25 (0.85 to 1.84) |
| Age at index date, years |  |  |  |  |
| <18 | 38 (3.6) | 139 (2.7) | 0.9 (-0.3 to 2.2) | 1.31 (0.91 to 1.88) |
| 18-≤28 | 78 (3.7) | 214 (2.1) | 1.6 (0.7 to 2.4) | 1.76 (1.35 to 2.29) |
| Calendar period at index date |  |  |  |  |
| 1987-2001 | 54 (3.6) | 176 (2.4) | 1.2 (0.2 to 2.2) | 1.50 (1.10 to 2.04) |
| 2002-2009 | 41 (3.9) | 102 (2.0) | 1.9 (0.6 to 3.1) | 1.98 (1.36 to 2.87) |
| 2010-2017 | 21 (3.6) | 75 (2.6) | 0.9 (-0.7 to 2.6) | 1.35 (0.82 to 2.22) |
| Educational attainment, years |  |  |  |  |
| 0-9 | 26 (3.6) | 85 (2.3) | 1.3 (-0.2 to 2.8) | 1.54 (0.87 to 2.72) |
| 10-12 | 56 (3.8) | 161 (2.3) | 1.5 (0.5 to 2.6) | 1.71 (1.22 to 2.42) |
| ≥13 | 30 (3.7) | 82 (2.2) | 1.5 (0.1 to 2.9) | 1.93 (1.08 to 3.45) |
| Missing | 4 (2.4) | 25 (3.0) | -0.6 (-3.2 to 2.0) | 0.76 (0.25 to 2.28) |
| History of autoimmune diseases |  |  |  |  |
| No | 102 (3.5) | 332 (2.3) | 1.3 (0.5 to 2.0) | 1.54 (1.23 to 1.93) |
| Yes | 14 (5.3) | 21 (3.7) | 1.6 (-1.6 to 4.9) | 2.00 (0.18 to 22.06) |
| CI: confidence interval; HR: hazard ratio; IBD: inflammatory bowel disease; IR: incidence rate; T1D: type 1 diabetes. | | | | |
| ^a^ Conditioned on the matching variables (birth year, sex, county of residence, and calendar year) and further adjusted for country of birth, educational attainment, and autoimmune diseases. | | | | |

**Table S8 Subgroup analyses of incident T1D in patients with CD, UC, IBD-U and their matched reference individuals in the cohort study**

| **Group** | **CD** | | | |  | | **UC** | | | | | |  | | **IBD-U** | | | | |
| --- | --- | --- | --- | --- | --- | --- | --- | --- | --- | --- | --- | --- | --- | --- | --- | --- | --- | --- | --- |
|  | **No. of events (IR, per 10,000 person-years)** | | **IR difference (95%CI), per 10,000 person-years** | **HR (95%CI) ^a^** |  | | **No. of events (IR, per 10,000 person-years)** | | | | **IR difference (95%CI), per 10,000 person-years** | **HR (95%CI) ^a^** |  | | **No. of events (IR, per 10,000 person-years)** | | | **IR difference (95%CI), per 10,000 person-years** | **HR (95%CI) ^a^** |
|  | **Patients** | **References** |  |  |  | | **Patients** | | | **References** |  |  |  | | **Patients** | | **References** |  |  |
| Sex |  |  |  |  |  | |  | | |  |  |  |  | |  | |  |  |  |
| Male | 22 (3.8) | 88 (3.1) | 0.7 (-1.0 to 2.4) | 1.18 (0.73 to 1.88) |  | | 52 (5.8) | | | 111 (2.5) | 3.2 (1.6 to 4.9) | 2.36 (1.68 to 3.32) |  | | 8 (3.9) | | 27 (2.7) | 1.1 (-1.7 to 4.0) | 1.32 (0.59 to 2.96) |
| Female | 11 (1.9) | 51 (1.9) | 0.0 (-1.2 to 1.3) | 0.99 (0.51 to 1.92) |  | | 16 (2.2) | | | 58 (1.7) | 0.5 (-0.6 to 1.7) | 1.35 (0.75 to 2.41) |  | | 7 (4.0) | | 18 (2.1) | 1.9 (-1.2 to 4.9) | 1.81 (0.73 to 4.50) |
| Age at index date, years | |  |  |  |  | | | |  |  |  |  | |  | |  |  |  |  |
| <18 | 13 (3.1) | 59 (2.9) | 0.3 (-1.6 to 2.1) | 1.04 (0.57 to 1.90) |  | | 20 (4.2) | | | 54 (2.3) | 1.9 (-0.0 to 3.8) | 1.79 (1.04 to 3.07) |  | | 5 (3.3) | | 26 (3.5) | -0.2 (-3.3 to 3.0) | 0.87 (0.32 to 2.37) |
| 18-≤28 | 20 (2.7) | 80 (2.3) | 0.4 (-0.9 to 1.7) | 1.14 (0.69 to 1.88) |  | | 48 (4.2) | | | 115 (2.1) | 2.1 (0.9 to 3.4) | 2.14 (1.51 to 3.03) |  | | 10 (4.3) | | 19 (1.7) | 2.6 (-0.2 to 5.4) | 2.12 (0.95 to 4.71) |
| Calendar period at index date | |  |  |  |  | | | |  |  |  |  | |  | |  |  |  |  |
| 1987-2001 | 14 (2.4) | 67 (2.4) | 0.0 (-1.4 to 1.4) | 0.96 (0.53 to 1.72) |  | | 37 (4.5) | | | 95 (2.4) | 2.1 (0.6 to 3.7) | 1.99 (1.35 to 2.94) |  | | 3 (2.4) | | 14 (2.3) | 0.1 (-2.9 to 3.0) | 1.08 (0.28 to 4.18) |
| 2002-2009 | 10 (2.7) | 43 (2.4) | 0.3 (-1.5 to 2.1) | 1.14 (0.57 to 2.29) |  | | 24 (4.5) | | | 44 (1.7) | 2.8 (0.9 to 4.6) | 2.88 (1.68 to 4.92) |  | | 7 (4.9) | | 15 (2.2) | 2.7 (-1.1 to 6.5) | 2.21 (0.87 to 5.60) |
| 2010-2017 | 9 (4.4) | 29 (2.9) | 1.5 (-1.6 to 4.5) | 1.35 (0.62 to 2.95) |  | | 7 (2.6) | | | 30 (2.3) | 0.3 (-1.8 to 2.4) | 1.18 (0.50 to 2.77) |  | | 5 (4.3) | | 16 (2.9) | 1.5 (-2.6 to 5.5) | 1.37 (0.46 to 4.07) |
| Educational attainment, years | |  |  |  | |  | |  | |  |  |  |  | |  | |  |  |  |
| 0-9 | 5 (1.8) | 31 (2.3) | -0.5 (-2.3 to 1.2) | 0.67 (0.19 to 2.35) |  | | 18 (5.4) | | | 38 (2.1) | 3.3 (0.7 to 5.9) | 3.38 (1.53 to 7.46) |  | | 3 (3.1) | | 16 (3.3) | -0.2 (-4.1 to 3.7) | 0.72 (0.15 to 3.48) |
| 10-12 | 18 (3.4) | 63 (2.5) | 0.9 (-0.8 to 2.6) | 1.15 (0.65 to 2.04) |  | | 28 (3.6) | | | 85 (2.3) | 1.3 (-0.1 to 2.8) | 1.97 (1.20 to 3.24) |  | | 10 (5.8) | | 13 (1.6) | 4.2 (0.5 to 7.9) | 3.65 (1.35 to 9.83) |
| ≥13 | 9 (3.3) | 35 (2.6) | 0.7 (-1.6 to 3.0) | 1.90 (0.69 to 5.20) |  | | 19 (4.4) | | | 32 (1.7) | 2.8 (0.7 to 4.8) | 3.81 (1.50 to 9.71) |  | | 2 (1.9) | | 15 (3.0) | -1.1 (-4.1 to 1.9) | 0.57 (0.12 to 2.71) |
| Missing | 1 (1.3) | 10 (2.7) | -1.4 (-4.5 to 1.7) | 0.61 (0.07 to 4.97) |  | | 3 (3.5) | | | 14 (3.2) | 0.3 (-4.0 to 4.6) | 0.96 (0.26 to 3.56) |  | | 0 (0.0) | | 1 (2.4) | -2.4 (-7.1 to 2.3) | NA |
| CD: Crohn's disease; CI: confidence interval; HR: hazard ratio; IBD-U: inflammatory bowel disease unclassified; IR: incidence rate; NA: not applicable; T1D: type 1 diabetes; UC: ulcerative colitis. | | | | | | | | | | | | | | | | | | | |
| ^a^ Conditioned on the matching variables (birth year, sex, county of residence, and calendar year) and further adjusted for country of birth, educational attainment, and autoimmune diseases. | | | | | | | | | | | | | | | | | | | |

**Table S9 Incident T1D in patients with IBD and their matched reference individuals in the cohort study, by phenotypes according to the Montreal Classification**

| **Group** | **T1D** | | | |
| --- | --- | --- | --- | --- |
|  | **No. of events (IR, per 10,000 person-years)** | | **IR difference (95%CI), per 10,000 person-years** | **HR (95%CI) ^a^** |
|  | **Patients** | **References** |  |  |
| IBD |  |  |  |  |
| PSC | 6 (13.4) | 3 (1.3) | 12.1 (1.3 to 22.9) | 13.02 (2.32 to 73.05) |
| Other extraintestinal manifestations | 5 (6.4) | 7 (1.9) | 4.5 (-1.2 to 10.3) | 5.10 (0.84 to 31.13) |
| CD |  |  |  |  |
| No location information | 10 (2.9) | 41 (2.4) | 0.4 (-1.5 to 2.4) | 1.16 (0.57 to 2.37) |
| L1, L3/LX (Ileal, ileocolonic or location not defined) | 21 (3.2) | 77 (2.4) | 0.8 (-0.7 to 2.2) | 1.31 (0.80 to 2.14) |
| L2 (Colonic) | 2 (1.3) | 21 (3.0) | -1.7 (-3.9 to 0.6) | 0.47 (0.10 to 2.08) |
| Perianal | 2 (3.0) | 14 (4.3) | -1.3 (-6.1 to 3.4) | 0.34 (0.04 to 2.65) |
| PSC | 0 (0.0) | 1 (3.7) | -3.7 (-11.0 to 3.6) | NA |
| Other extraintestinal manifestations | 2 (5.7) | 3 (1.8) | 3.9 (-4.3 to 12.2) | 5.11 (0.41 to 63.69) |
| UC |  |  |  |  |
| No extent information ^b^ | 22 (4.7) | 59 (2.5) | 2.1 (0.1 to 4.2) | 1.85 (1.11 to 3.08) |
| E1/E2 (Proctitis, left-sided colitis) | 6 (2.0) | 20 (1.4) | 0.6 (-1.1 to 2.3) | 1.59 (0.61 to 4.17) |
| E3 (Extensive colitis) | 14 (4.8) | 33 (2.4) | 2.5 (-0.2 to 5.2) | 1.99 (1.03 to 3.85) |
| EX (Extent not defined) | 26 (4.7) | 57 (2.1) | 2.6 (0.7 to 4.4) | 2.35 (1.45 to 3.80) |
| PSC | 5 (15.4) | 2 (1.2) | 14.2 (0.6 to 27.8) | 18.77 (2.11 to 167.20) |
| Other extraintestinal manifestations | 2 (6.9) | 3 (2.2) | 4.7 (-5.1 to 14.6) | NA |
| IBD-U |  |  |  |  |
| PSC | 1 (14.5) | 0 (0.0) | 14.5 (-13.9 to 42.8) | NA |
| Other extraintestinal manifestations | 1 (7.0) | 1 (1.5) | 5.5 (-8.4 to 19.4) | NA |
| CD: Crohn’s disease; CI: confidence interval; E: Extent; HR: hazard ratio; IBD: inflammatory bowel disease; IR: incident rate; L: location; NA: not appliable; PSC: primary sclerosing cholangitis; T1D: type 1 diabetes; UC: ulcerative colitis. | | | | |
| ^a^ Conditioned on the matching variables (birth year, sex, county of residence, and calendar year) and further adjusted for country of birth, educational attainment, and autoimmune diseases. | | | | |
| ^b^ No extent information refers to patients who were diagnosed with UC before the introduction of ICD-10 in 1997. | | | | |

**Table S10 Sensitivity analyses of incident T1D in patients with IBD and their matched reference individuals in the cohort study**

| **Group** | **T1D** | | | |
| --- | --- | --- | --- | --- |
|  | **No. of events (IR, per 10,000 person-years)** | | **IR difference (95%CI), per 10,000 person-years** | **HR (95%CI) ^a^** |
|  | **Patients** | **References** |  |  |
| Discard the first year of follow-up from analysis |  |  |  |  |
| Overall IBD | 94 (3.2) | 315 (2.2) | 1.0 (0.3 to 1.7) | 1.42 (1.12 to 1.79) |
| CD | 23 (2.1) | 123 (2.4) | -0.3 (-1.2 to 0.7) | 0.87 (0.55 to 1.36) |
| UC | 59 (3.9) | 152 (2.1) | 1.8 (0.8 to 2.9) | 1.93 (1.41 to 2.63) |
| IBD-U | 12 (3.4) | 40 (2.4) | 1.0 (-1.0 to 3.1) | 1.32 (0.68 to 2.55) |
| Discard the first three years of follow-up from analysis |  |  |  |  |
| Overall IBD | 76 (3.0) | 254 (2.1) | 0.9 (0.2 to 1.6) | 1.41 (1.08 to 1.83) |
| CD | 17 (1.8) | 94 (2.1) | -0.3 (-1.3 to 0.7) | 0.84 (0.49 to 1.41) |
| UC | 47 (3.6) | 128 (2.0) | 1.5 (0.5 to 2.6) | 1.79 (1.27 to 2.53) |
| IBD-U | 12 (4.0) | 32 (2.3) | 1.8 (-0.7 to 4.2) | 1.66 (0.84 to 3.29) |
| Only primary diagnosis for T1D |  |  |  |  |
| Overall IBD | 92 (2.9) | 324 (2.1) | 0.8 (0.1 to 1.4) | 1.37 (1.08 to 1.73) |
| CD | 24 (2.1) | 125 (2.2) | -0.2 (-1.1 to 0.7) | 0.91 (0.59 to 1.42) |
| UC | 58 (3.6) | 156 (2.0) | 1.6 (0.6 to 2.6) | 1.87 (1.37 to 2.55) |
| IBD-U | 10 (2.6) | 43 (2.3) | 0.3 (-1.5 to 2.0) | 1.00 (0.50 to 2.02) |
| At least two diagnoses for T1D |  |  |  |  |
| Overall IBD | 92 (2.9) | 291 (1.9) | 1.0 (0.4 to 1.6) | 1.52 (1.20 to 1.93) |
| CD | 25 (2.2) | 111 (2.0) | 0.2 (-0.8 to 1.1) | 1.07 (0.69 to 1.66) |
| UC | 57 (3.5) | 142 (1.8) | 1.7 (0.7 to 2.7) | 1.99 (1.45 to 2.74) |
| IBD-U | 10 (2.6) | 38 (2.1) | 0.5 (-1.2 to 2.3) | 1.17 (0.58 to 2.37) |
| Individuals with available educational attainment |  |  |  |  |
| Overall IBD | 112 (3.7) | 328 (2.3) | 1.5 (0.7 to 2.2) | 1.65 (1.33 to 2.05) |
| CD | 32 (3.0) | 129 (2.5) | 0.5 (-0.6 to 1.6) | 1.18 (0.79 to 1.74) |
| UC | 65 (4.2) | 155 (2.1) | 2.1 (1.1 to 3.2) | 2.13 (1.58 to 2.87) |
| IBD-U | 15 (4.0) | 44 (2.4) | 1.6 (-0.6 to 3.7) | 1.52 (0.84 to 2.76) |
| Additionally censored diabetic nephropathy and pancreatic conditions from the analysis ^b^ | | | |  |
| Overall IBD | 116 (3.7) | 351 (2.3) | 1.4 (0.7 to 2.1) | 1.59 (1.28 to 1.96) |
| CD | 33 (2.9) | 138 (2.5) | 0.4 (-0.7 to 1.4) | 1.12 (0.76 to 1.65) |
| UC | 68 (4.2) | 169 (2.2) | 2.1 (1.0 to 3.1) | 2.02 (1.51 to 2.70) |
| IBD-U | 15 (3.9) | 44 (2.4) | 1.5 (-0.6 to 3.7) | 1.52 (0.84 to 2.76) |
| Additionally censored at date of first IBD-related surgery | |  |  |  |
| Overall IBD | 94 (3.8) | 346 (2.3) | 1.5 (0.7 to 2.3) | 1.63 (1.29 to 2.08) |
| CD | 25 (3.1) | 137 (2.5) | 0.5 (-0.7 to 1.8) | 1.18 (0.76 to 1.85) |
| UC | 56 (4.3) | 165 (2.1) | 2.1 (1.0 to 3.3) | 2.04 (1.48 to 2.80) |
| IBD-U | 13 (4.0) | 44 (2.4) | 1.6 (-0.7 to 3.9) | 1.56 (0.82 to 2.96) |
| Additionally censored at date of first steroids prescription after IBD diagnosis ^c^ | | |  |  |
| Overall IBD | 13 (4.9) | 100 (2.3) | 2.6 (-0.1 to 5.3) | 2.26 (1.12 to 4.56) |
| CD | 5 (5.4) | 38 (2.6) | 2.8 (-2.0 to 7.5) | 2.04 (0.67 to 6.22) |
| UC | 6 (4.9) | 40 (2.0) | 3.0 (-1.0 to 7.0) | 3.15 (1.05 to 9.45) |
| IBD-U | 2 (4.2) | 22 (2.9) | 1.2 (-4.6 to 7.1) | 1.79 (0.32 to 10.02) |
| Additionally censored at date of first biologics prescription after IBD diagnosis ^c^ | | | |  |
| Overall IBD | 35 (3.9) | 113 (2.2) | 1.7 (0.3 to 3.1) | 1.68 (1.13 to 2.50) |
| CD | 14 (4.8) | 46 (2.6) | 2.2 (-0.4 to 4.9) | 1.66 (0.88 to 3.12) |
| UC | 15 (3.4) | 44 (1.8) | 1.6 (-0.2 to 3.4) | 1.95 (1.05 to 3.62) |
| IBD-U | 6 (3.7) | 23 (2.5) | 1.2 (-2.0 to 4.3) | 1.23 (0.46 to 3.26) |
| CD: Crohn's disease; CI: confidence interval; HR: hazard ratio; IBD(-U): inflammatory bowel disease (unclassified); IR: incidence rate; T1D: type 1 diabetes; UC: ulcerative colitis. | | | | |
| ^a^ Conditioned on the matching variables (birth year, sex, county of residence, and calendar year) and further adjusted for country of birth, educational attainment, and autoimmune diseases. | | | | |
| ^b^ Further censored the analysis at date of diagnosis with diabetes insipidus, nephrogenic diabetes, pancreatitis, pancreatic insufficiency, or pancreatic cancer. | | | | |
| ^c^ Restricted the analysis to individuals with an index date of January 2006 or later. | | | | |

**Table S11 Characteristics of patients w**ith IBD and their IBD-free full siblings in the cohort study

|  | **Full siblings** | **Patients with IBD** | **Subtypes of IBD, n(%)** | | |
| --- | --- | --- | --- | --- | --- |
|  |  |  | **CD** | **UC** | **IBD-U** |
| N | 22201 | 14511 | 5250 | 7174 | 2087 |
| Age at index date, years ^a^ |  |  |  |  |  |
| Mean ± SD | 18.1 ± 6.1 | 19.3 ± 5.3 | 18.9 ± 5.2 | 19.8 ± 5.2 | 18.6 ± 5.5 |
| Median (IQR) | 18.8 (14.2-22.8) | 19.8 (15.9-23.3) | 19.3 (15.3-23.0) | 20.3 (16.6-23.7) | 19.0 (15.3-22.9) |
| <18 | 8586 (38.7) | 5468 (37.7) | 2175 (41.4) | 2394 (33.4) | 899 (43.1) |
| 18-≤28 | 13615 (61.3) | 9043 (62.3) | 3075 (58.6) | 4780 (66.6) | 1188 (56.9) |
| Female | 10790 (48.6) | 6641 (45.8) | 2528 (48.2) | 3148 (43.9) | 965 (46.2) |
| Born in Nordic country | 20928 (94.3) | 13854 (95.5) | 4963 (94.5) | 6884 (96.0) | 2007 (96.2) |
| Calendar period at index date ^a^ |  |  |  |  |  |
| 1987-2001 | 6692 (30.1) | 4427 (30.5) | 1625 (31.0) | 2413 (33.6) | 389 (18.6) |
| 2002-2009 | 7690 (34.6) | 4967 (34.2) | 1792 (34.1) | 2491 (34.7) | 684 (32.8) |
| 2010-2017 | 7819 (35.2) | 5117 (35.3) | 1833 (34.9) | 2270 (31.6) | 1014 (48.6) |
| Educational attainment, years |  |  |  |  |  |
| 0-9 | 4834 (21.8) | 3557 (24.5) | 1376 (26.2) | 1640 (22.9) | 541 (25.9) |
| 10-12 | 6973 (31.4) | 6588 (45.4) | 2343 (44.6) | 3323 (46.3) | 922 (44.2) |
| ≥13 | 3161 (14.2) | 3998 (27.6) | 1357 (25.9) | 2031 (28.3) | 610 (29.2) |
| Missing | 7233 (32.6) | 368 (2.5) | 174 (3.3) | 180 (2.5) | 14 (0.7) |
| History of autoimmune diseases | 1040 (4.7) | 1296 (8.9) | 657 (12.5) | 391 (5.5) | 248 (11.9) |
| Follow-up time, years |  |  |  |  |  |
| Median (IQR) | 14.1 (9.0-20.3) | 14.5 (9.3-20.7) | 14.7 (9.3-20.9) | 15.2 (9.8-21.0) | 11.8 (8.1-17.5) |
| 0-0.9 | 143 (0.6) | 62 (0.4) | 23 (0.4) | 32 (0.5) | 7 (0.3) |
| 1-4.9 | 900 (4.1) | 408 (2.8) | 144 (2.7) | 177 (2.5) | 87 (4.2) |
| 5-9.9 | 5744 (25.9) | 3682 (25.4) | 1324 (25.2) | 1664 (23.2) | 694 (33.3) |
| 10-19.9 | 9551 (43.0) | 6331 (43.6) | 2264 (43.1) | 3116 (43.4) | 951 (45.6) |
| ≥20 | 5863 (26.4) | 4028 (27.8) | 1495 (28.5) | 2185 (30.5) | 348 (16.7) |
| CD: Crohn’s disease; IBD(-U): inflammatory bowel disease (unclassified); IQR: interquartile range; SD: standard deviation; UC: ulcerative colitis. | | | | | |
| ^a^ Index date: date of IBD diagnosis for patients with IBD, and date of selection for their IBD-free full siblings. | | | | | |

**Table S12** Incident T1D in patients with IBD and their IBD-free full siblings in the cohort study

|  | **No. of events (IR, per 10,000 person-years)** | | **IR difference (95%CI), per 10,000 person-years** | **HR (95%CI)** | |
| --- | --- | --- | --- | --- | --- |
|  | **Patients** | **Full siblings** |  | **Model 1 ^a^** | **Model 2 ^b^** |
| Overall IBD | 82 (3.6) | 85 (2.5) | 1.1 (0.2 to 2.1) | 1.39 (1.01 to 1.91) | 1.44 (0.97 to 2.15) |
| CD | 22 (2.7) | 30 (2.4) | 0.2 (-1.2 to 1.6) | 0.98 (0.55 to 1.75) | 0.85 (0.35 to 2.06) |
| UC | 50 (4.4) | 43 (2.5) | 1.8 (0.4 to 3.3) | 1.75 (1.14 to 2.67) | 1.90 (1.12 to 3.24) |
| IBD-U | 10 (3.6) | 12 (2.9) | 0.7 (-2.1 to 3.5) | 1.17 (0.49 to 2.78) | 0.97 (0.29 to 3.25) |
| CD: Crohn's disease; CI: confidence interval; HR: hazard ratio; IBD(-U): inflammatory bowel disease (unclassified); IR: incidence rate; T1D: type 1 diabetes; UC: ulcerative colitis. | | | | | |
| ^a^ Conditioned on family identifier and adjusted for birth year, sex, county of residence, and calendar year. | | | | | |
| ^b^ Further adjusted for country of birth, educational attainment, and autoimmune diseases. | | | | | |

**Table S13 Clinical phenotypes of patients with IBD in the case-control study, n (%)**

|  | **Subtypes of IBD** | | |
| --- | --- | --- | --- |
|  | **CD** | **UC** | **IBD-U** |
| Extraintestinal manifestations at index date |  |  |  |
| PSC | 162 (0.6) | 752 (1.6) | 194 (1.5) |
| Other extraintestinal manifestations | 1536 (6.0) | 1862 (3.9) | 896 (7.0) |
| Montreal Classification CD at index date |  |  |  |
| No location information | 6434 (25.0) | - | - |
| L1, L3/LX (Ileal, ileocolonic or location not defined) | 15576 (60.4) | - | - |
| L2 (Colonic) | 3773 (14.6) | - | - |
| Perianal | 1335 (5.2) | - | - |
| Montreal Classification UC at index date |  |  |  |
| No extent information ^a^ | - | 11234 (23.2) | - |
| E1/E2 (Proctitis, left-sided colitis) | - | 13860 (28.6) | - |
| E3 (Extensive colitis) | - | 7483 (15.5) | - |
| EX (Extent not defined) | - | 15829 (32.7) | - |
| CD: Crohn’s disease; E: Extent; IBD(-U): inflammatory bowel disease (unclassified); L: location; PSC: primary sclerosing cholangitis; UC: ulcerative colitis. | | | |
| ^a^ No extent information refers to patients who were diagnosed with UC before the introduction of ICD-10 in 1997. | | | |

**Table S14 Prior T1D in patients with** IBD in the case-control study

|  | No. individuals with/without T1D | | OR (95%CI) | |
| --- | --- | --- | --- | --- |
|  | Patients | Controls | Model 1 ^a^ | Model 2 ^b^ |
| Overall IBD | 1018/85983 | 3496/427558 | 1.45 (1.35 to 1.56) | 1.36 (1.26 to 1.46) |
| CD | 264/25519 | 984/126807 | 1.33 (1.16 to 1.53) | 1.17 (1.02 to 1.35) |
| UC | 530/47876 | 1903/237983 | 1.39 (1.26 to 1.53) | 1.36 (1.23 to 1.50) |
| IBD-U | 224/12588 | 609/62768 | 1.84 (1.58 to 2.15) | 1.63 (1.39 to 1.91) |
| CD: Crohn's disease; CI: confidence interval; IBD(-U): inflammatory bowel disease (unclassified); IR: incidence rate; T1D: type 1 diabetes; OR: odds ratio; UC: ulcerative colitis.  ^a^ Conditioned on the matching variables (birth year, sex, county of residence, and calendar year).  ^b^ Further adjusted for country of birth, educational attainment, and autoimmune diseases. | | | | |

**Table S15 Subgroup analyses of prior T1D in patients with IBD in the case-control study**

|  | **No. of individuals with/without T1D** | | **OR (95%CI)** | |
| --- | --- | --- | --- | --- |
|  | **Patients** | **References** | **Model 1 ^a^** | **Model 2 ^b^** |
| Sex |  |  |  |  |
| Male | 572/44372 | 1958/220706 | 1.46 (1.33 to 1.60) | 1.40 (1.28 to 1.54) |
| Female | 446/41611 | 1538/206852 | 1.44 (1.30 to 1.60) | 1.29 (1.16 to 1.44) |
| Age at index date, years |  |  |  |  |
| <18 | 40/7041 | 151/35175 | 1.33 (0.94 to 1.88) | 1.11 (0.77 to 1.59) |
| 18-<40 | 256/32921 | 984/163262 | 1.29 (1.12 to 1.48) | 1.17 (1.02 to 1.35) |
| 40-<60 | 297/25961 | 937/128889 | 1.58 (1.38 to 1.80) | 1.45 (1.27 to 1.65) |
| ≥60 | 425/20060 | 1424/100232 | 1.49 (1.34 to 1.67) | 1.47 (1.32 to 1.65) |
| Calendar period at index date |  |  |  |  |
| 1987-2001 | 182/31528 | 549/157043 | 1.66 (1.40 to 1.96) | 1.54 (1.30 to 1.82) |
| 2002-2009 | 388/27612 | 1352/137354 | 1.43 (1.28 to 1.60) | 1.38 (1.23 to 1.55) |
| 2010-2017 | 448/26843 | 1595/133161 | 1.40 (1.26 to 1.55) | 1.28 (1.15 to 1.43) |
| Educational attainment, years |  |  |  |  |
| 0-9 | 272/19826 | 933/96839 | 1.40 (1.17 to 1.67) | 1.30 (1.09 to 1.55) |
| 10-12 | 454/35965 | 1366/171239 | 1.52 (1.34 to 1.73) | 1.41 (1.24 to 1.60) |
| ≥13 | 215/22494 | 771/114721 | 1.38 (1.13 to 1.68) | 1.30 (1.07 to 1.59) |
| Missing | 77/7698 | 426/44759 | 1.60 (1.15 to 2.23) | 1.48 (1.05 to 2.08) |
| History of autoimmune diseases |  |  |  |  |
| No | 715/74509 | 2874/396501 | 1.35 (1.24 to 1.47) | 1.35 (1.24 to 1.47) |
| Yes | 303/11474 | 622/31057 | 1.26 (0.98 to 1.62) | 1.33 (1.03 to 1.72) |
| CI: confidence interval; IBD: inflammatory bowel disease; OR: odds ratio; T1D: type 1 diabetes | | | | |
| ^a^ Conditioned on the matching variables (birth year, sex, county of residence, and calendar year). | | | | |
| ^b^ Further adjusted for country of birth, educational attainment, and autoimmune diseases. | | | | |

**Table S16 Subgroup analyses of prior T1D in patients with IBD in the case-control study, by phenotypes according to** the Montreal Classification

|  | **No. of individuals with/without T1D** | | **OR (95%CI)** | |
| --- | --- | --- | --- | --- |
|  | **Patients** | **Controls** | **Model 1 ^a^** | **Model 2 ^b^** |
| IBD |  |  |  |  |
| PSC | 31/1077 | 47/5421 | 3.34 (2.11 to 5.29) | 2.97 (1.83 to 4.81) |
| Other extraintestinal manifestations | 103/4191 | 253/20944 | 2.03 (1.61 to 2.56) | 1.60 (1.23 to 2.08) |
| CD |  |  |  |  |
| No location information | 18/6416 | 106/31891 | 0.84 (0.51 to 1.39) | 0.69 (0.42 to 1.16) |
| L1, L3/LX (Ileal, ileocolonic or location not defined) | 198/15378 | 715/76397 | 1.37 (1.17 to 1.61) | 1.22 (1.04 to 1.44) |
| L2 (Colonic) | 48/3725 | 163/18519 | 1.46 (1.05 to 2.01) | 1.32 (0.95 to 1.84) |
| Perianal | 20/1315 | 65/6535 | 1.54 (0.93 to 2.56) | 1.50 (0.90 to 2.51) |
| PSC | 4/158 | 10/785 | 1.97 (0.62 to 6.27) | 1.89 (0.57 to 6.25) |
| Other extraintestinal manifestations | 31/1505 | 94/7477 | 1.65 (1.09 to 2.50) | 1.31 (0.81 to 2.13) |
| UC |  |  |  |  |
| No extent information ^c^ | 69/11165 | 168/55635 | 2.06 (1.55 to 2.73) | 1.92 (1.44 to 2.55) |
| E1/E2 (Proctitis, left-sided colitis) | 141/13719 | 644/67987 | 1.09 (0.90 to 1.30) | 1.10 (0.91 to 1.32) |
| E3 (Extensive colitis) | 97/7386 | 340/36705 | 1.42 (1.13 to 1.78) | 1.38 (1.10 to 1.73) |
| EX (Extent not defined) | 223/15606 | 751/77656 | 1.49 (1.28 to 1.73) | 1.46 (1.26 to 1.70) |
| PSC | 16/736 | 32/3684 | 2.55 (1.39 to 4.70) | 2.20 (1.16 to 4.17) |
| Other extraintestinal manifestations | 43/1819 | 103/9093 | 2.06 (1.44 to 2.95) | 1.76 (1.19 to 2.62) |
| IBD-U |  |  |  |  |
| PSC | 11/183 | 5/952 | 10.85 (3.77 to 31.24) | 12.93 (4.04 to 41.44) |
| Other extraintestinal manifestations | 29/867 | 56/4374 | 2.60 (1.65 to 4.09) | 1.77 (1.06 to 2.95) |
| CD: Crohn’s disease; CI: confidence interval; E: Extent; IBD(-U): inflammatory bowel disease (unclassified); L: location; T1D: type 1 diabetes; OR: odds ratio; PSC: primary sclerosing cholangitis; UC: ulcerative colitis. | | | | |
| ^a^ Conditioned on the matching variables (birth year, sex, county of residence, and calendar year). | | | | |
| ^b^ Further adjusted for country of birth, educational attainment, and autoimmune diseases. | | | | |
| ^c^ No extent information refers to patients who were diagnosed with UC before the introduction of ICD-10 in 1997. | | | | |

**Table S17 Sensitivity analyses of prior T1D in patients with IBD in the** case-control study

|  | **No. of individuals with/without T1D** | | **OR (95%CI)** | |
| --- | --- | --- | --- | --- |
|  | **Patients** | **References** | **Model 1 ^a^** | **Model 2 ^b^** |
| Excluding T1D experienced during the last year before index date |  |  |  |  |
| Overall IBD | 809/85781 | 3209/427155 | 1.26 (1.16 to 1.36) | 1.17 (1.08 to 1.27) |
| CD | 201/25465 | 905/126690 | 1.10 (0.95 to 1.29) | 0.97 (0.83 to 1.13) |
| UC | 420/47778 | 1740/237764 | 1.20 (1.08 to 1.34) | 1.17 (1.05 to 1.31) |
| IBD-U | 188/12538 | 564/62701 | 1.67 (1.41 to 1.97) | 1.48 (1.25 to 1.76) |
| Excluding T1D experienced during the last three years before index date |  |  |  |  |
| Overall IBD | 663/85447 | 2716/425909 | 1.22 (1.12 to 1.33) | 1.14 (1.04 to 1.24) |
| CD | 166/25350 | 780/126209 | 1.07 (0.90 to 1.27) | 0.93 (0.79 to 1.11) |
| UC | 340/47609 | 1444/237214 | 1.17 (1.04 to 1.32) | 1.14 (1.01 to 1.29) |
| IBD-U | 157/12488 | 492/62486 | 1.61 (1.34 to 1.93) | 1.43 (1.18 to 1.72) |
| CD: Crohn’s disease; CI: confidence interval; IBD(-U): inflammatory bowel disease (unclassified); T1D: type 1 diabetes; OR: odds ratio; UC: ulcerative colitis. | | | | |
| ^a^ Conditioned on the matching variables (birth year, sex, county of residence, and calendar year). | | | | |
| ^b^ Further adjusted for country of birth, educational attainment, and autoimmune diseases. | | | | |

| **Table S18 Prior T1D in patients with IBD and their IBD-free full siblings in the case-control study** | | | | |
| --- | --- | --- | --- | --- |
|  | **No. of individuals with/without T1D** | | **OR (95%CI)** | |
|  | **Patients** | **Full siblings** | **Model 1 ^a^** | **Model 2 ^b^** |
| Overall IBD | 621/56219 | 918/106720 | 1.32 (1.18 to 1.47) | 1.32 (1.18 to 1.49) |
| CD | 159/16716 | 246/31448 | 1.27 (1.02 to 1.58) | 1.33 (1.05 to 1.67) |
| UC | 324/31410 | 507/59753 | 1.23 (1.06 to 1.43) | 1.21 (1.03 to 1.42) |
| IBD-U | 138/8093 | 165/15519 | 1.67 (1.31 to 2.13) | 1.74 (1.32 to 2.29) |
| CD: Crohn's disease; CI: confidence interval; IBD(-U): inflammatory bowel disease (unclassified); IR: incidence rate; T1D: type 1 diabetes; OR: odds ratio; UC: ulcerative colitis. | | | | |
| ^a^ Conditioned on family identifier and adjusted for birth year, sex, county of residence, and calendar year. | | | | |
| ^b^ Further adjusted for country of birth, educational attainment, and autoimmune diseases. | | | | |

**References:**

1. Lu S, Gong J, Tan Y, Liu D. Epidemiologic Association between Inflammatory Bowel Diseases and Type 1 Diabetes Mellitus: a Meta-Analysis. Journal of Gastrointestinal and Liver Diseases. 2020;29(3):407-13.

2. Conrad N, Misra S, Verbakel JY, Verbeke G, Molenberghs G, Taylor PN, et al. Incidence, prevalence, and co-occurrence of autoimmune disorders over time and by age, sex, and socioeconomic status: a population-based cohort study of 22 million individuals in the UK. Lancet. 2023;401(10391):1878-90.

3. Jasser‐Nitsche H, Bechtold‐Dalla Pozza S, Binder E, Bollow E, Heidtmann B, Lee‐Barkley YH, et al. Comorbidity of inflammatory bowel disease in children and adolescents with type 1 diabetes. Acta Paediatr. 2021;110(4):1353-8.

4. Ghersin I, Khateeb N, Katz LH, Daher S, Shamir R, Assa A. Comorbidities in adolescents with inflammatory bowel disease: findings from a population-based cohort study. Pediatric Research. 2020;87(7):1256-62.

5. Park S-W, Kim TJ, Lee JY, Kim ER, Hong SN, Chang DK, et al. Comorbid immune-mediated diseases in inflammatory bowel disease: a nation-wide population-based study. Alimentary Pharmacology & Therapeutics. 2019;49(2):165-72.

6. Burisch J, Jess T, Egeberg A. Incidence of Immune-Mediated Inflammatory Diseases Among Patients With Inflammatory Bowel Diseases in Denmark. Clinical Gastroenterology and Hepatology. 2019;17(13):2704-12.e3.

7. Bar Yehuda S, Axlerod R, Toker O, Zigman N, Goren I, Mourad V, et al. The Association of Inflammatory Bowel Diseases with Autoimmune Disorders: A Report from the epi-IIRN. J Crohns Colitis. 2019;13(3):324-9.

8. Halling ML, Kjeldsen J, Knudsen T, Nielsen J, Hansen LK. Patients with inflammatory bowel disease have increased risk of autoimmune and inflammatory diseases. World J Gastroenterol. 2017;23(33):6137-46.

9. Virta LJ, Kolho KL. The risk of contracting pediatric inflammatory bowel disease in children with celiac disease, epilepsy, juvenile arthritis and type 1 diabetes--a nationwide study. J Crohns Colitis. 2013;7(1):53-7.

10. Kappelman MD, Galanko JA, Porter CQ, Sandler RS. Association of paediatric inflammatory bowel disease with other immune-mediated diseases. Arch Dis Child. 2011;96(11):1042-6.

11. Forss A, Clements M, Bergman D, Roelstraete B, Kaplan G, Gilaad, Myrelid P, et al. A nationwide cohort study of the incidence of inflammatory bowel disease in Sweden from 1990 to 2014. Alimentary Pharmacology & Therapeutics. 2022;55(6):691-9.

12. Mouratidou N, Malmborg P, Järås J, Sigurdsson V, Sandström O, Fagerberg UL, et al. Identification of Childhood-Onset Inflammatory Bowel Disease in Swedish Healthcare Registers: A Validation Study. Clinical Epidemiology. 2022;Volume 14:591-600.

13. Olén O, Erichsen R, Sachs MC, Pedersen L, Halfvarson J, Askling J, et al. Colorectal cancer in ulcerative colitis: a Scandinavian population-based cohort study. The Lancet. 2020;395(10218):123-31.
